# Safe and Sustainable by Design (SSbD) Synthesis of Food and Personal Care Ingredients by Mechanochemistry

**DOI:** 10.1002/cssc.202502491

**Published:** 2026-05-04

**Authors:** Rubén Solórzano‐Rodríguez, Nicolas Fantozzi, Andrea Casagrande, Christos M. Chatzigiannis, Corentin Bordier, Pietro Rando, Evelina Colacino

**Affiliations:** ^1^ ICGM Univ Montpellier, CNRS, ENSCM Montpellier France; ^2^ Virumaa Kolledž Tallinn University of Technology Ida‐Viru maakond Estonia; ^3^ CRB Benelux BV Maastricht The Netherlands

**Keywords:** active ingredients, biomass transformation, green chemistry metrics, mechanochemistry, polymeric jars

## Abstract

The preparation of perillartine, a potent marketed sweetener, and *N*‐tert‐butyl‐α‐phenylnitrone (PBN), a most commonly used free‐radical spin‐trap used in personal care formulation, endowed with antioxidant, neuroprotective, and anti‐aging properties, was studied by vibrating and planetary ball‐milling. The reactivity in polymeric jars, such as PTFE and in‐house made polyoxymethylene (POM), was evaluated and compared to the most commonly used stainless steel and zirconium oxide milling media. This investigation revealed an insightful correlation while also accounting for the energy involved in each transformation. Assessment of the processes by green chemistry metrics and tools (*e.g.*, Chem21, DOZN 3.0 and EcoScale) showed that mechanochemical approaches offer a Safe and Sustainable by Design (SSbD) approach also complying with the IUPAC guiding principles of a responsible chemistry. The reduced solvent consumption, the simplified purification procedures, and lower associated environmental and safety hazards are strongly aligned with the green chemistry principles.

## Graphical abstract

A Safe and Sustainable by Design (SSbD) mechanochemical approach was used to prepare a marketed sweetener from a biomass derivative used in flavoring industry. A personal care ingredient was also synthesized. The study also evaluated and compared the reactivity in of polymeric jars with that in of common conventional stainless steel or and zirconium oxide milling media. In‐house‐made, fluorine‐free polyoxymethylene (POM) is proposed as a valid alternative to PTFE.

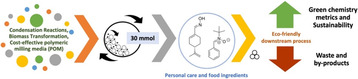

## 1 Introduction

The need for environmentally sustainable chemical processes has become a critical focus for modern science and industry, driving significant interest in green chemistry approaches. Traditionally, chemical processes rely on hazardous solvents, require substantial energy inputs, and produce significant amounts of waste, posing challenges to both environmental sustainability and economic efficiency. The transition to greener chemical methods supports the United Nations’ 17 Sustainable Development Goals (UN SDGs) [1], promoting practices that minimize waste, improve resource efficiency, and enhance safety. In particular, mechanochemistry has been identified as a transformative technology that directly contributes to achieving several of these goals (the link between mechanochemistry and the UN SDGs appeared for the first time in the literature in [2]; the concept was further expanded in [3]). IUPAC highlighted mechanochemistry as a key technology for sustainable chemical synthesis [4], offering a solvent‐free alternative to traditional processes and a powerful approach to implement the 12 principles of green chemistry [5, 6]. In pharmaceutical and fine chemical manufacturing, solvents typically account for 80%–90% of the reaction mass [7], 75% of the energy consumption, and up to 50% of the global warming potential, posing a substantial issue that needs to be addressed [8]. In contrast, mechanochemical methods exhibit improved green metrics [9, 10], including lower energy requirements, reduced waste generation, and improved safety by avoiding toxic solvents. When appropriately designed and assessed, such mechanochemical processes are Safe and Sustainable by Design (SSbD). Additionally, in our believe, they comply with the framework of “IUPAC guiding principles of responsible chemistry” [11] recently unveiled and calling on scientists, educators, industry professionals, policymakers, and future chemists to direct their work toward addressing the pressing challenges facing humanity.

**SCHEME 1:**
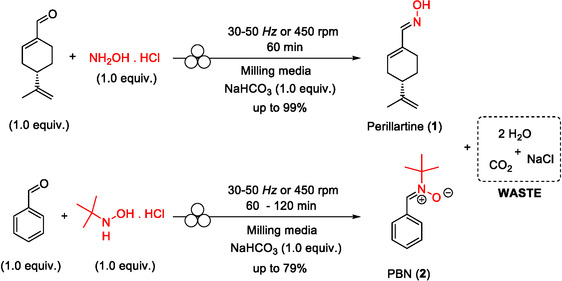
Mechanochemical preparation of (*S*)‐(‐)‐perillartine (**1**) and *N*‐*tert*‐butyl‐α‐phenylnitrone (PBN, **2**).

One particularly illustrative example where mechanochemical processes and green chemistry principles work in synergy is the synthesis of high‐value compounds derived from natural or renewable resources. Here, we describe the mechanochemical synthesis of (*S*)‐(‐)‐perillartine (**1**) and *N*‐*tert*‐butyl‐α‐phenylnitrone (**2**, PBN) from inexpensive, readily available precursors, using protocols that minimize waste generation and are SSbD‐compliant (Scheme 1). (*S*)‐(‐)‐Perillartine, also known as perilla sugar, is a potent, Food and Drug Administration (FDA)‐approved [12] semisynthetic sweetener (approximately 2000 times sweeter than sucrose) that has attracted attention due to its potential biological activities. It is derived from *Perilla frutescens*, a crop widely distributed in East Asian countries and commonly used not only in cuisine, but also in traditional medicine due to its diverse pharmacological properties [13, 14, 15, 16, 17]. The synthesis of perillartine typically involves the condensation reaction of perillaldehyde, with hydroxylamine hydrochloride [18, 19]. Perillaldehyde is a natural compound extracted from *P. frutescens* and used as food additive for flavoring and in perfumery to add spiciness.

Recently, green alternative synthetic protocols were reported using electrochemical [20] and chemoenzymatic [21] procedures. Despite their efficiency, these methods require uncommon experimental setups limiting their broader applicability.

**FIGURE 1:**
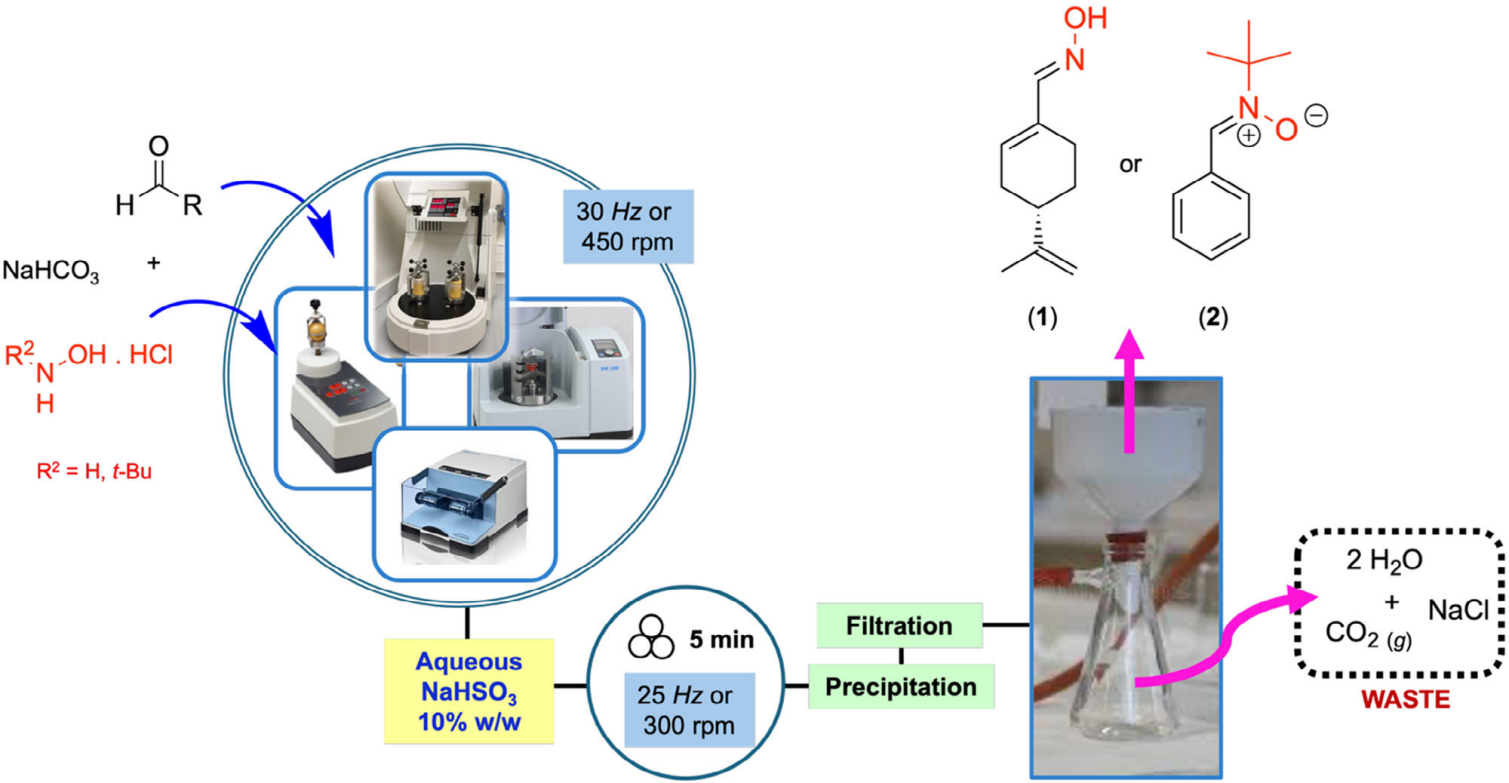
Downstream and eco‐friendly process for recovering (*S*)‐(‐)‐perillartine (**1**) and *N*‐*tert*‐butyl‐α‐phenylnitrone (PBN, **2**), with no need of organic solvents. Images of the milling equipment were reproduced by kind permission of (a) Retsch (GmbH, Germany) for vibrating ball‐mill (MM400) and PM100 (planetary mill), and (b) Fritsch (GmbH, Germany) for vibrating ball‐mill (P23) and P7 Classic line (planetary mill).

We previously described the mechanochemical preparation of oximes as key intermediates for the Beckman rearrangement to amides [22, 23]. Aldoxymes were also prepared by a mechanochemical condensation reaction of arylaldehydes with hydroxylamine [24]. Herein, we extended the study to the mechanochemical preparation of aldoxime (*S*)‐(‐)‐Perillartine (**1**) by ball‐milling. Beyond the simplicity of the experimental setup, the reactions do not require anhydrous conditions or the use of organic solvents, including in the downstream process to recover the final product (Figure 1).

Similarly, *N*‐*tert*‐butyl‐*α*‐phenylnitrone (**2**, PBN) is a well‐known free‐radical spin trap that exhibits neuroprotective and antiaging properties [25, 26, 27, 28, 29, 30, 31]. Due to its antioxidant properties and its ability to mitigate oxidative stress‐related conditions, it has attracted interest from both academia and industry. Its main application lies in personal care, and it is registered as a cosmetic ingredient in CosIng, the European Commission's database of cosmetic substances and ingredients [32].

Commonly used solvent‐based methods to synthesize nitrones involve mainly i) condensation reactions of *N*‐monosubstituted hydroxylamines, ii) oxidation of different substrates (*N*,*N*‐disubstituted hydroxylamines, secondary amines, *N*‐alkyl‐α‐amino acids, imines and isoxazolidines), iii) reactions of oximes with electrophiles, and iv) reaction of nitro compounds with aldehydes under reductive conditions [33]. Specifically, synthetic procedures for PBN reported in the literature, either solution‐based or microwave‐assisted processes, rely on the first approach [34, 35, 36, 37]. A preliminary report on the mechanochemical synthesis of nitrones was first investigated by one of us [38].

In parallel to long‐lasting and ongoing work targeting the preparation of Active Pharmaceutical Ingredients (APIs) by different mechanochemical methods (both in batch and in continuous) [23, 39, 40, 41, 42], we wish to expand the application of mechanochemical processing to other value‐added molecules to new market sectors, such as food and personal care, where sustainability is still poorly addressed. Therefore, we report the mechanochemical preparation of the sweetener perillartine from a naturally occurring, and abundant renewable product of commercial interest, the flavoring agent *Perilla frutescens* and expand further the preparation of the antioxidant *N*‐*tert*‐butyl‐α‐phenylnitrone (**2**, PBN), under different processing conditions. This includes the use of polyoxymethylene (POM) as grinding media, quite unexplored in organic synthesis, expanding the range of mechanochemical processing conditions by introducing new metal and fluorine‐free milling media (POM), offering a more sustainable, healthier, and less expensive option compared to polytetrafluoroethylene (PTFE) jars. Indeed, per‐ and polyfluoroalkyl substances (PFAS) are progressively recognized as persistent environmental pollutants, and PTFE falls within this chemical class. By providing a fluorine‐free option, polyoxymethylene (POM) is consistent with a proactive Safe and Sustainable by Design framework, thereby reducing potential future risks associated with persistent pollutants. In addition, POM represents also suitable alternative to stainless steel (SS), as it can prevent or reduce the likelihood of residual metal contamination arising from abrasion of the milling media.

The mechanochemical protocol herein developed is robust and transferable across different common milling platforms (vibratory and planetary), which is essential for its practical adoption by the wider chemical community. The study is completed by an in‐depth analysis of the process's full eco‐footprint, which is a key contribution that moves beyond simple reaction yields. The comprehensive and quantitative green assessment of the entire workflow includes the often‐neglected downstream processing by an array of green chemistry metrics calculations [9, 10], using Chem21 [43], DOZN 3.0 [41, 44, 45], and Eco‐scale [46] tools. The scores are compared with the corresponding solution‐based preparation methods, validating the mechanochemical method's alignment with SSbD principles and the IUPAC Guiding Principles of Responsible chemistry [11].

## 2 Results and Discussion

The mechanochemical synthesis of (*S*)‐(‐)‐perillartine (**1**) and PBN (**2**) was explored under different mechanical stresses (vibrating and planetary ball‐milling) and milling media. Both compounds were synthesized through a direct condensation reaction between equimolar amounts of the corresponding aldehyde and hydroxylamine hydrochloride salt, in the presence of one equivalent of NaHCO_3_ (Scheme 1). Taking advantage of their low solubility in water, (S)‐(‐)‐perillartine (**1**) and PBN (**2**) were readily recovered from the crude reaction mixture using three simple steps: precipitation with aqueous sodium bisulfite (10% w/w), filtration, and drying. No organic solvents were required, even during downstream processing, making the entire workflow—from synthesis to recovery—both straightforward and eco‐friendly (Figure 1).

To evaluate the influence of different grinding media on the outcome of the reaction, jars in stainless steel (SS), zirconium oxide, and two polymeric materials such as polytetrafluoroethylene (PTFE) and polyoxymethylene (POM) were compared. SS and zirconium oxide are well‐established grinding media due to their chemical resistance and availability. However, the release of metal impurities due to the abrasion of hard grinding media cannot be excluded, especially when SS is used [47, 48]. On the other hand, polymeric vessels have recently emerged as cost‐effective, alternatives to the conventional metallic and ceramic jars. Poly(methyl methacrylate) (PMMA) is the most commonly used polymeric material especially for in situ monitoring purposes [49].

However, its chemical compatibility and mechanical resistance are somehow limited, and in some cases not so effective as other harder and denser milling media [50] for covalent bond forming reactions (for a comparative example on the use of PMMA *vs* SS/ZrO_2_ grinding media in the synthesis of an Active Pharmaceutical Ingredient (Dantrolene): a) synthesis in SS or ZrO_2_ jars [51]; b) synthesis in SS or ZrO_2_ jars [52]). Ertalyte jars were also used for metal‐free mechanochemical oxidation reactions [53].

The choice of the polymeric jars can be tailored to meet specific mechanochemical process requirements, such as chemical and thermal resistance, transparency to UV‐Vis, X‐ray radiation or Raman beam, or mechanical resistance to abrasion [54]. PTFE excels in chemical and thermal resistance; however, PTFE vessels tend to deform due to their inherent softness. POM, although less resistant than PTFE in terms of chemical and thermal stability, allows for stronger, stiffer vessel construction, minimizing deformation during milling and potentially inducing different mechanical stresses [55, 56, 57]. POM jars proved to be resistant over repeated runs, with no visual abrasion or deformation. Contrarily to hard material such as SS or ZrO_2_, for which the amount of residual “leached” metal can be measured directly from the crude mixture (e.g., by ICP‐MS methods), leachate analyses for POM vessels are beyond the scope of this process‐study. Indeed, it would be necessary to manufacture “ad hoc” in situ monitoring devices, able to detect “online” the gaseous byproducts generated by the degradation of POM under mechanochemical processing. The technical advancements and experimental setups available nowadays in the field of “real‐time in situ/in operando monitoring of mechanochemical reactions,” make use of PXRD diffractometers, RAMAN probes, or thermal cameras. These techniques are not suitable for detecting the gaseous byproducts generated by POM depolymerization (in case this would occur). Whether at some point, other “analytical facilities” coupled to milling devices were to become available in the future, still, suitable analytical methods should be disclosed. Indeed, the main byproduct that could be generated by degradation of POM (due to heat, UV exposure, or chemical attack) comes from its depolymerization back to its monomer units (formaldehyde), especially when POM is heated above ~230°C or burned [55, 56, 57]. We exclude that these extreme conditions can be reached in our processing conditions, ruling out already the hypothesis of the degradation of POM vessels. Other secondary degradation products such as formic acid and carbon monoxide (CO) could also be formed through oxidation of formaldehyde (if any). CO_2_ and MeOH (most rarely, it is formed in trace amounts depending on the presence of moisture and additives) could also be formed when the POM material is subjected to even higher temperatures or flames. Again, we exclude the formation of these secondary degradation byproducts of POM (and generated from formaldehyde) during the mechanochemical processing conditions reported in our manuscript.

One advantage of polymeric jars is their compatibility with various ball (or bead) materials, which allows to isolate (understand) the effects of the ball composition while keeping the jar material constant. For each of the mechanochemical processing conditions explored (Tables 1 and 2), a freely accessible online tool [59] was used to calculate the kinetic energy of the milling balls transferred to the molecules upon impact, distinguishing between the total energy (*E*_total,_ in kJ) transferred to the reaction mixture during milling, and the energy per single impact (*E*_impact_, in mJ) between the ball and the molecules. The total mass (total *m*_balls_) was also considered, due to its contribution to the total kinetic energy (*E*_total_).

**TABLE 1:** Selection of data for mechanochemical processing conditions using different grinding media or their combinations, in the preparation of (*S*)‐(‐)‐perillartine (**1**).

Entry^a^	Jar material	Ball material	Number of balls	Diameter (Ø mm)	*Total m*_balls_ (g)^a^	*E*_total_ (kJ)	*E*_impact_ (mJ)	Yield (%)
1^b^	SS	SS	2	7	2.8	21.3	9.9	99
2^b^	SS	SS	15	5	7.6	57.3	3.6	73
3^b^	PTFE	SS	15	5	7.6	57.3	3.6	95
4^b^	**POM**	SS	15	5	7.6	57.3	3.6	**96** a
5^b^	ZrO_2_	ZrO_2_	15	5	6.2	46.9	2.9	91
6^b^	**POM**	ZrO_2_	15	5	6.2	46.9	2.9	**98**
7^b^^,^ c	PTFE	ZrO_2_	15	5	6.2	46.9	2.9	90
8^b^^,^ c	PTFE	SS	2	7	2.8	21.3	9.9	89
9^b^^,^ c	**POM**	SS	2	7	2.8	21.3	9.9	**98**
10^d^	SS	SS	10	5	5.1	38.6	3.6	95
11^d^	PTFE	Stainless steel	8	5	4.1	30.9	3.6	98
12^d^	PTFE	ZrO_2_	8	5	3.3	25.3	2.9	96
13^e^	ZrO_2_	ZrO_2_	30	5	12.4	18.6	6.0	98
14^e^	SS	SS	30	5	15.2	22.7	7.3	88^f^
15^c^^,^ e	ZrO_2_	ZrO_2_	110	5	45.5	107.4	11.3	90^g^
16^c^^,^ e	SS	SS	110	5	55.7	131.0	13.8	91^g^

a *Reaction scale:* 4.0 mmol except for entry 4 (2.0 mmol). Equimolar amounts of reagents were always used. 100% conversion was always obtained except for entry 2. The product was always recovered by precipitation using 10% aqueous NaHSO_3_. The weight for each type of milling ball used for the experiments is given in Table S1. The electrical energy consumption associated with the milling experiments is reported in Table S2. Legend : SS = Stainless steel.

b *Reaction conditions*: Horizontal vibrating ball‐mill (MM400) 30 Hz, 1 h, 10 mL jar. The geometry of the milling vessel was identical, including for the in house‐made POM jar.

c K_2_HPO_4_ was used as base (1 equiv.), at 30 Hz, 1 h in a horizontal vibrating ball‐mill (MM400).

d Vertical vibrating ball‐mill (P23): 50 Hz, 1 h.

e Planetary ball‐mill (P7 classic: entries 13 and 14; PM100: entries 15 and 16): 450 rpm, 1 h. The reaction scale was 4.0 mmol (entries 13 and 14) or 30 mmol (entries 15 and 16).

f 10% of residual aldehyde was detected in the crude mixture (determined by ^1^H NMR analyses).

g Corrected yield calculated from isolated product using qNMR, as (*S*)‐perillaldehyde used in these batches contained a 10% of cuminaldehyde [58] as an impurity.

**TABLE 2:** Selection of mechanochemical processing conditions using different grinding media or their combinations, in the preparation of *N*‐*tert*‐butyl‐α‐phenylnitrone (PBN, **2**).

Entry^a^	Jar material	Balls material	Number of balls	Diameter (Ø mm)	*Total m*_balls_ (g)	*E*_total_ (kJ)	*E*_impact_ (mJ)	Yield (%)
1^b^	SS	SS	2	7	2.8	42.6	9.9	100 [38]
2^c^	SS	SS	15	5	6.2	114.6	3.6	10
3^c^	**ZrO**_**2**_	ZrO_2_	15	5	6.2	93.8	2.9	**76**
4^c^	PTFE	SS	15	5	7.6	114.6	3.6	45
5^c^	POM	SS	15	5	7.6	114.6	3.6	45
6^c^	**POM**	ZrO_2_	15	5	6.2	93.8	2.9	**65**
7^d^	SS	SS	10	5	5.1	38.6	3.6	74
8^d^	PTFE	SS	8	5	4.1	30.9	3.6	67
9^d^	PTFE	ZrO_2_	8	5	3.3	25.3	2.9	0
10^e^	**ZrO**_**2**_	ZrO_2_	30	5	12.4	18.6	5.6	**79**
11^e^	SS	SS	30	5	15.2	22.8	7.3	10

a *Reaction scale:* 4.0 mmol, except for entry 7 (2.0 mmol). Equimolar amounts of reagents were always used. 100% conversion was always obtained except for entry 2. The product was always recovered by precipitation in aqueous NaHSO_3_ 10% w/w. The weight for each type of milling ball used for the experiments is given in Table S1. The electrical energy consumption associated with the milling experiments is reported in Table S2. *Legend*: SS = Stainless steel.

b Reaction conditions reported previously [38].

c Horizontal vibrating ball‐mill (MM400) 30 Hz, 2 h, 10 mL jar. The geometry of the milling vessel was identical, including for the in‐house‐made POM jar.

d Vertical vibrating ball‐mill (P23): 50 Hz, 1 h, 5 mL (entries 8 and 9) or 10 mL (entry 7) jar.

e Planetary ball‐mill (P7 classic): 450 rpm, 1 h using K_2_HPO_4_ as base (1.0 equiv.). The reaction scale was 4.0 mmol (12 mL jar).

Therefore, in the case of the mechanochemical preparation of (*S*)‐(‐)‐perillartine (**1**), and already since the first trial, (*S*)‐(‐)‐perillartine (**1**) was obtained in 99% yield by milling equimolar amounts of the reactants at 30 Hz during 1 h in a SS jar (10 mL) with 2 x 7 mm balls of the same material (Table 1, entry 1). No further optimization was needed, highlighting the intrinsic efficiency of mechanochemistry in facilitating condensation reactions. However, when the experiment was repeated by replacing the 2 × 7 mm balls (entry 1) by 15 × 5 mm (entry 2), the yield dropped to 73%, due to uncomplete conversion of the aldehyde.

The lower amount of energy delivered to the reactants during each single impact (*E*_impact_ = 3.6 mJ) could slow down the transformation rate into the product [60], in favor of other side reactions, such as hydration reaction of the starting aldehyde, as a consequence of a higher cumulative total energy (*E*_total_ = 57.3 kJ). However, heating effects at microscopic and/or macroscopic levels, produced during friction and impacts, cannot be excluded. In the case of SS, having higher thermal conductivity (*λ*) (14.9 W/mK) compared to zirconia (in the range 1.7–2.7 W/mK) [61], the hydration reaction of aldehyde could be favored, due to a higher heating dissipation promoting a rise in bulk temperature.

On the contrary, in ZrO_2_, its significantly lower thermal conductivity potentially limiting heat dissipation, the condensation reaction is favored by local temperature rises. This trend was also confirmed when ZrO_2_ (entry 5) was used instead of SS (entry 2): the yield increased to 91%, despite the lower cumulative total energy (*E*_total_ = 46.9 kJ vs. 57.3 kJ in entry 2), as a consequence of the lower density of ZrO_2_ in comparison to stainless steel [50]. This observation suggests that higher energy input does not necessarily correlate with higher yield, providing valuable insights into the distinct reactivity profiles of mechanochemical reactions.

Along the same line, other comparative experiments in a planetary ball‐mill (entries 13 and 14), differing only in the nature of the milling media, showed that zirconia outperformed stainless steel, affording a 98% yield versus 88%, respectively. ^1^H NMR analyses of the latter revealed a 10% residual amount of unreacted aldehyde. In the best conditions for the planetary mill, up to *ca.* 4.5 g of (*S*)‐(‐)‐perillartine (**1**), could be obtained in one run. To better understand the influence of the milling media and isolate the effect of the jar material, hard vessels were replaced with softer polymeric jars made of PTFE or POM. The milling conditions were adjusted to maintain a constant cumulative total energy (*E*_total_) across experiments (entries 3–4, 5–7, and 1, 8, 9), by varying the type (SS or ZrO_2_), size (*Ø* = 5 or 7 mm), and number (2, 8, 10, or 15) of milling balls (Table 1).

The results showed that the outcome of the reaction depended primarily on the jar material. POM consistently delivered higher yields compared to ZrO_2_ (entries 6 vs. 5), stainless steel (entries 4 vs. 2), and PTFE (entries 9 vs. 8), regardless of the ball material used. Yields obtained in POM were even comparable to those obtained in SS jars under optimized conditions (entries 9 vs. 1).

Chemical parameters need also to be considered. Therefore, the influence of the base was also examined. Therefore, for comparative purposes with NaHCO_3_, bases such as NaOH and Na_2_CO_3_ were also tested, using identical mechanochemical reaction and work‐up, as those outperforming for the synthesis of perillartine **2** using POM jars (Table 1, entry 6). In the case of NaOH as a base, no product was formed upon precipitation (0% yield). The crude was an orange paste, very different from the white powder usually obtained for perillartine (**2**). When Na_2_CO_3_ was used, the isolated yield increased to 78%, but it was still lower than the benchmark experiment (Table 1, entry 6), delivering 98% yield. These experiments confirmed, furthermore, the validity of the mechanochemical method, with NaHCO_3_ being the optimal base for this transformation.

High yields were maintained when NaHCO_3_ was replaced by K_2_HPO_4_ (entries 7–9, 15, and 16), regardless of reaction scale (4.0 or 30 mmol) or milling conditions. Notably, when *E*_total_ was kept constant at 21.3 kJ (entries 8 and 9) and milling was performed at 30 Hz in either POM or PTFE jars, POM again outperformed PTFE (98% vs. 89% yield, respectively).

The reaction was also successfully scaled up to 30 mmol in a planetary ball mill (entries 15 and 16), giving comparable yields regardless of the milling media used (SS or ZrO_2_). From a process development perspective, this is advantageous, as it circumvents potential limitations related to the cost or availability of ZrO_2_ vessels for large‐scale applications, and potential constraint in machinery.

Although K_2_HPO_4_ sometimes gave slightly lower yields than NaHCO_3_, it offers an important operational benefit: unlike NaHCO_3_, it does not release CO_2_ during the reaction. This eliminates the risk of pressure buildup in closed milling vessels, which can become a critical issue at larger scales.

In general, (*S*)‐(‐)‐perillartine (**1**) was obtained in very good to excellent yields (up to 99%) and with full selectivity toward the *E*‐regioisomer. The pure product was recovered by avoiding the use of organic solvents and chromatographic purifications, by a straightforward precipitation/filtration sequence of operations. The reaction was efficient regardless of the type of milling media or combinations thereof, milling frequency used (30 or 50 Hz), scale (4.0 or 30 mmol), or type of milling device used (Table 1). Even though the nature of the polymeric material constituting the jars [54] can affect both conversions and yields [51, 52], herein the reaction can also be facilitated by its favorable rheology, as the starting aldehyde is a liquid, absorbed onto the solid reagents. As soon as the reaction proceeds to completion, a solid cake is formed, recovered as fine, dry powder after the aqueous work‐up (Figure 1).

Similarly, the mechanochemical preparation of *N*‐tert‐butyl‐α‐phenylnitrone (PBN, **2**; *Z*‐regioisomer) was investigated (Scheme 1 and Table 2), focusing on the influence of the milling jar composition—including combinations of different milling materials—on reaction productivity. In the case of polymeric jars made of PTFE or POM, balls composed of either SS or zirconia were used. The results were evaluated in terms of mechanochemical processing conditions and compared to those previously reported for a vibrating ball mill (Table 2, entry 1) [38], where stainless steel was used as milling media. The rationale was to identify an alternative milling setup that avoids residual metal contamination, particularly for applications involving human use or consumption, as previously demonstrated in the mechanochemical synthesis of (*S*)‐(‐)‐perillartine (**1**). At 30 Hz, in reaction conditions set to provide higher stress frequency (S_F_) [62, 63] enabling more impacts per unit of time (entry 2), with a 3‐fold total cumulative energy (*E*_total_ = 114.6 vs. 42.6 kJ in entry 1), the yield dropped drastically (10%) and the conversion of starting materials remained low. This trend confirmed the observation and considerations made so far for the preparation of (*S*)‐(‐)‐perillartine (**1**) (Table 1, entries 1 and 2). By keeping constant the total cumulative energy (*E*_total_ = 114.6 kJ, entries 2, 4, and 5), the reaction output was strictly dependent on the material used for the milling jar. Thus, PBN (**2**) yield increased to 45% by replacing SS jar by “softer” polymer vessels, probably due to the lower thermal conductivity diminishing the extent of hydration reaction of the aldehyde, such as POM or PTFE. At 30 Hz, among the possible combinations, PTFE/POM with SS/ZrO_2_ balls, POM/ZrO_2_ combination was the best, confirming the potential of this polymeric material as a valuable alternative to the commonly used PTFE. Also, Teflon is a fluoropolymer, and any potential residue from leaching could be problematic—especially in applications involving human consumption. From an environmental perspective as well, this is a concern, given that fluoropolymers like Teflon are highly persistent in the environment.

At a lower total cumulative energy (in the range *ca.* 31–39 kJ, entries 7 and 8), yields were increased up to 74%, as the milling frequency was 50 Hz, for PTFE/SS ball combination or in SS (both jar and balls). However, for PTFE, by replacing SS balls (entry 8) with ZrO_2_ ones (entry 9), despite a relatively small difference in the total cumulative energy [Δ(*E*_total_) = 5.6 kJ], reagents were not converted (0% yield).

Comparable yields were obtained when ZrO_2_ milling media was used to prepare PBN (**2**), in both vibrating and planetary ball‐mill (76% and 79% yield, entries 3 and 10, respectively), with ZrO_2_ becoming the material of choice in this last case. Indeed, even if the benchmark experiment in the vibrating ball‐mill (entry 1) [38] delivered quantitative yield of PBN (**2**) in SS, the same reaction in the planetary ball‐mill was not efficient (10% yield, entry 11), despite the little difference in the total cumulative energy [Δ(*E*_total_) = 4.6 kJ, entries 10 vs. 11)]. This could be due to a more favorable balance between mechanical stress and less important macroscopic thermal effects for reactions carried out in ZrO_2_ (entries 2 vs. 3, and 10 vs. 11).

As expected, for this transformation, the output of the reactions was mainly dependent on the interplay between technical (e.g., type of ball‐mill and milling media, milling jar volume and shape, size and number of milling balls, ball to mass ratio, and filling degree) and process parameters (e.g., operating frequency and reaction time) [9, 62, 63], confirming, once more, that the optimization of a mechanochemical reaction is multifactorial and the reactivity of the system needs fine tuning of several variables. Independently on the yields and the processing conditions used, the *Z*‐regioisomer was always obtained with full selectivity.

Similarly to (*S*)‐(‐)‐perillartine (**1**), PBN (**2**) was easily recovered without the use of organic solvents (Figure 1). The melting point measured for PBN (**2**) after work‐up by precipitation of the crude in aqueous NaHSO_3_ (10%) was 70°C–71°C, closely matching the value reported in the literature (71°C–72°C), whether the product was recovered by recrystallization from hexane [64], or by evaporation in vacuo from dichloromethane [38]. This fact suggested that the same solid form could have been formed in all cases, aspect of particular interest especially when dealing with drugs [65], or any substance for which the stability of crystalline properties over time and/or under varying environmental conditions is essential [66].

### 2.1 Comparative Greenness Assessment of Mechanochemical vs. Solution‐Based Processes

Several methods were previously reported for the solution‐based synthesis of (*S*)‐(‐)‐perillartine (**1**) and *N*‐*tert*‐butyl‐α‐phenylnitrone (PBN, **2**), briefly described below for context and comparative purposes. Mostly, (*S*)‐(‐)‐perillartine (**1**) was prepared by dissolving hydroxylamine hydrochloride and sodium carbonate in water, followed by the gradual addition of perillaldehyde. After 2 h reaction, a liquid–liquid extraction was performed using ethyl acetate. Further purification using column chromatography with cyclohexane/ethyl acetate afforded perillartine in 62% yield [18, 19]. Another method, although similar, did not report yield and therefore could not be directly compared [67]. More recently, electrochemical [20] and chemoenzymatic [21] synthesis of (*S*)‐(‐)‐perillartine (**1**) were described. In the first, (*S*)‐(‐)‐perillartine (**1**) was obtained after 10 h in 90% yield through the generation of NH_2_OH by the electrocatalytic reduction of KNO_3_ in water, using a multilayered Zn nanosheet catalyst as electrode, needed for the addition of perillaldehyde under acidic conditions. On the other hand, the chemoenzymatic method relies on the oxidation of perillyl alcohol by *Acetobacter* spp., followed by in situ condensation of perillaldehyde with an excess of aqueous hydroxylamine hydrochloride (1.5 equiv.). This approach afforded (*S*)‐(‐)‐perillartine (**1**) in 96% yield after 48 h, after purification by column chromatography.

When compared with the mechanochemical method presented herein in Table 1, the solution‐based protocols already exhibit several limitations. The classical preparation of aldoximes [19], although using water as solvent, requires a larger solvent volume and results in significantly lower 58% yield impacting negatively the overall green metrics. The more recent electrochemical [20] and chemoenzymatic [21] processes afforded comparable yields (96% and *ca*. 90%, respectively) to the mechanochemical process (Table 1). However, these methods rely on specialized electrochemical setups or bacterial cultures, which are often less accessible in standard synthetic laboratories. In contrast, the mechanochemical approach employed here requires the use of common milling equipment, making it more practical and easier to implement in most research and industrial settings. Moreover, the mechanochemical protocol avoids the use of metals as catalysts and provides similar or even superior yields, while drastically reducing reaction times from 10–48 h  to 1 h.

In the case of *N*‐*tert*‐butyl‐α‐phenylnitrone (PBN, **2**), most of the solution‐based methods for its preparation rely on the use of slight excess of pyrrolidine as a base and dichloromethane as the solvent. Under these conditions, PBN (**2**) was reported to be formed after 15 min [35, 36].

By substituting dichloromethane with a less harmful mixture of water and methanol, reaction time increased to 6 h. Solventless procedures were also reported in the literature. In one example, microwave‐assisted activation was used to afford PBN (**2**) in 86% yield after 2 min of microwave irradiation at about 140°C, after purification by column chromatography [34]. In another case, PBN (**2**) was obtained in 90% yield by stirring a mixture of benzaldehyde and *N*‐*tert*‐butyl hydroxylamine hydrochloride in the presence of a 2.5‐fold excess of magnesium oxide under an inert atmosphere for 24 h, after recrystallization from highly volatile solvents (VOS), namely a harmful dichloromethane/diethyl ether mixture [37].

Although these solution‐based protocols reported the formation of PBN (**2**) in a shorter time (2–15 min vs. 60–120 min for the mechanochemical methods), they rely on either the use of energy‐intensive microwave heating or the use of dichloromethane, a toxic solvent recently banned from the majority of industrial and commercial applications due to its environmental persistence and risks to human health [68, 69]. In contrast, the proposed mechanochemical protocols (Table 2) avoid the use of solvents, including in the downstream process (Figure 1), no heating is required, and only aqueous waste, not containing harmful compounds, is generated while maintaining a high yield.

However, beyond these qualitative considerations, already harnessing the advantages of mechanochemistry to conduct organic syntheses [70], a quantitative assessment of the eco‐footprint of mechanochemical methods vs. solution‐based approaches is always needed.

Indeed, mechanochemistry has emerged as a powerful approach for enhancing green metrics in chemical processes [9, 10] and their sustainability profiles [45, 71], in comparison with the solution‐based counterparts. Herein, the green chemistry metrics of the chemical transformations described above (Scheme 1 and Tables 1 and 2) were calculated using Chem21 [43], while the DOZN [41, 44, 45] and EcoScale [46] toolkits evaluate each process based on the 12 Principles of Green Chemistry and the reaction efficiency and downstream process, respectively. The developed mechanochemical methods were compared against the corresponding solution‐based processes. The protocols selected for (*S*)‐(‐)‐perillartine (**1**) [18] and *N*‐*tert*‐butyl‐α‐phenylnitrone (PBN, **2**) [36] synthesis were chosen based on similarities in chemical reagents and work‐up procedures.

#### 2.1.1 Green Metrics assessment by Chem21 tool [43]

The assessment by Chem21 tool combines both quantitative and qualitative indicators to evaluate the sustainability of a process (Table 3). Quantitative factors include yield, selectivity, and key metrics such as atom economy (AE) [73], reaction mass efficiency (RME) [74], optimum efficiency (OE) [43], process mass intensity (PMI) [75], and *E*‐factor [75]. These parameters were calculated for (*S*)‐(‐)‐perillartine (**1**) and *N*‐*tert*‐butyl‐α‐phenylnitrone (PBN, **2**), synthesized by both mechanochemical and solution‐based methods, to compare the environmental impact of each approach (see Table 3 and Excel spreadsheets in the Supporting Information). Notably, the inclusion of OE offers an advantage over AE and RME, as it accounts for variations inherent to different chemical processes. Optimum efficiency (OE) metric enables direct comparative assessment across disparate reaction classes [43]. A capability not consistently afforded by metrics such as atom economy (AE) or reaction mass efficiency (RME). This discrepancy arises from the fact that certain transformations are intrinsically atom‐ or mass‐efficient, whereas others are not, rendering OE a more broadly applicable and discriminating metric for evaluating the efficiency of diverse synthetic methodologies [69].

**TABLE 3:** Comparison of green chemistry metrics by Chem21 toolkit for solution‐based *vs.* mechanochemical methods in the preparation of (*S*)‐(‐)‐perillartine (**1**) and *N*‐*tert*‐butyl‐α‐phenylnitrone (PBN) (**2**).

Solution synthesis vs. mechanochemical conditions						
**METRICS** a ^,^ b	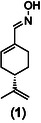			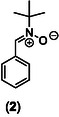		
**AE**	40.0	vs.	54.4	58.5	vs.	56.1
**RME**	24.4	vs.	53.1	54.2	vs.	36.5
**OE** c	60.9	vs.	97.6	92.7	vs.	65.0
**PMI**	65.1	vs.	21.3	185.5	vs.	29.9
***E*‐factor**	64.1	vs.	20.3	184.5	vs.	28.9
**Solvent**	**H_2_O**		**No solvent**	**CH**_**2**_ **Cl**_**2**_		**No solvent**
**Work‐up,** **recovery**	**Liquid extraction** , **Recryst** . ( **EtOAc** , ***c‐*C**_**6**_ **H**_**12**_ )		**Filtration**	**Filtration** ( **EtOAc** , **SiO**_**2**_ )		**Filtration** ( **NaHSO**_**3**_ **aq.** )
**Health and safety**						
	H410, H411		n.a.	H330, H350, H370, H372		n.a.
	H301, H351, H371, H373, H400			H301, H360FD, H371, H373, H401, H412		

a The optimum value for AE, RME, and OE is 100%; for *E*‐factor, the optimum value is 0, with PMI = E factor +1 [72]. When comparing two processes, the one with the lowest PMI will be the greenest.

b Experimental protocols used for metrics calculations. Methods in solution: for (**1**), reference [18] was used; for (**2**), reference [36] was used. Mechanochemical methods: for (**1**), Table 1 entry 6; for (**2**), Table 2, entry 6.

c $OE=\frac{RME}{AE}\times 100$. *Legend*: **Green flag** = preferred; **amber flag** = acceptable with some concerns; **red flag** = undesirable; n.a. = not applicable.

In addition to numerical metrics, Chem21 evaluates qualitative factors such as energy efficiency, safety, and potential environmental and health hazards associated with the chemicals and processes used. For each factor, a clear visual scoring system is assigned: green for “preferred,” amber for “acceptable with some concerns,” and red for “undesirable,” ensuring an intuitive and transparent evaluation of sustainability.

For the mechanochemical synthesis of (*S*)‐(‐)‐perillartine (**1**), all quantitative metrics were improved compared to the solution‐based procedure [18]: AE was increased due to the use of a lower molecular weight base (sodium hydrogen carbonate instead of sodium carbonate); RME was improved due to stoichiometric use of reagents; OE was close to the optimal value of 100%; and PMI and *E*‐factor, which take into account solvents and work‐up procedures, were substantially reduced due to the low solvent requirements inherent to the mechanochemical method, including the downstream process. In terms of qualitative evaluation, the nature of the solvents used for the work‐up process (water, green flag vs. cyclohexane, amber flag), as well as the quantity of reagents used (stoichiometric, amber flag vs. excess, red flag), further favored the mechanochemical method over the solution‐based one (also see Supporting Information).

In contrast, for *N*‐*tert*‐butyl‐α‐phenylnitrone (PBN, **2**), the quantitative assessment revealed differences in the values of AE, RME, and OE (Table 3).

This result arises from the fact that these metrics only consider the reagents involved (i.e., their molecular weight and stoichiometry) and not the solvents. Specifically, AE metric for the solution‐based method was slightly better because a lower molecular weight base was used (pyrrolidine instead of sodium hydrogen carbonate), while RME and OE were favored for the solution method due to a higher yield (97% vs. 65% for the mechanochemical method in Table 2, entry 6). However, assessing the greenness of a process solely on these metrics might be biased. When solvents were included in the calculations, an approximately 6‐fold decrease in PMI and *E*‐factor values could be observed in favor of the mechanochemical process. The qualitative evaluation also supported this finding, highlighting the use of hazardous solvents (dichloromethane) and silica during the purification step, which account for more than the 86% of the total PMI for PBN (**2**), in comparison with the mechanochemical method selected for the calculations, using POM/ZrO_2_ as milling media (65% yield, Table 2, entry 6). In this case, even if the yield of the mechanochemical process was lower (65% vs. 97% for the solution process [18]), the metrics accounting for the work‐up procedures were still better (*c.f.*, Excel spreadsheet in the Supporting Information), harmful solvents were not used, and the value of PMI for the mechanochemical process remained lower compared to the PMI calculated of the solution‐based protocol. These quantitative evaluations support the potential of mechanochemistry as a “Safe and Sustainable by Design” (SSbD) approach for organic synthesis and emphasize the need for green chemistry metrics to account for downstream processing. Indeed, solvent use during work‐up remains one of the most significant challenges undermining the benefits of mechanochemical reactions. Moreover, the safety and the hazards for each process need to be considered. Ideally, environmental impact should be evaluated through Life Cycle Assessment (LCA); however, such data are not always available or accessible, even for solution‐based methods at an industrial scale [71].

#### 2.1.2 Assessment by DOZN 3.0 toolkit

DOZN 3.0 toolkit evaluates chemical processes based on the 12 Principles of Green Chemistry [76], grouping them into three main categories: i) *Group 1*: Improved Resource Use, which considers principles 1, 2, 7, 8, 9, and 11; ii) *Group 2*: Increased Energy Efficiency, which includes principle 6 and evaluates deviations from ambient conditions regarding temperature and pressure; and iii) *Group 3*: Reduced Human and Environmental Hazards, which includes principles 3, 4, 5, 10, and 12, assessing waste generation, its severity, and the hazard profile of the final product. For each group, an individual score is calculated and then normalized to an aggregate value (referred to as “aggregate score”) and calculated by the average of the individual scores of each green chemistry principle. It provides a quantitative measure of the overall greenness of a process, with values approaching zero indicating greater sustainability. When comparing two processes, the one with the lower aggregate score is considered the more environmentally benign.

**FIGURE 2:**
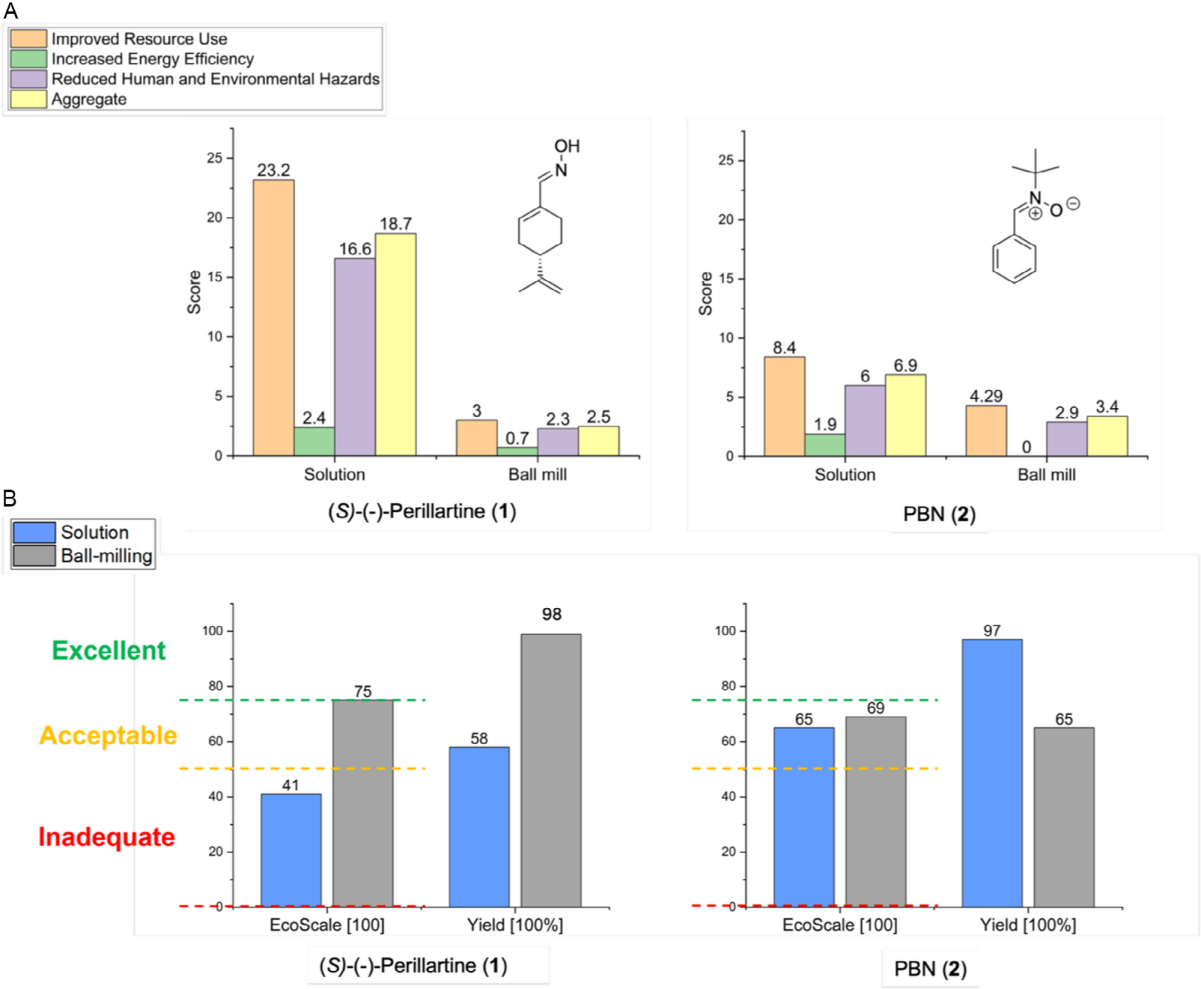
Comparison of solution‐based vs. mechanochemical synthesis for (*S*)‐(‐)‐perillartine (**1**) and *N*‐*tert*‐butyl‐α‐phenylnitrone (PBN, **2**). (A) By DOZN 3.0 Quantitative Scoring tool [44, 45]. Legend: *Group 1* (Improved Resource Use—**orange**), *Group 2* (Increased Energy Efficiency—**green**), *Group 3* (Reduced Human and Environmental Hazards—**violet**), and Aggregate score—**yellow**; (B) by EcoScale tool [46]. Legend: excellent (≥75), acceptable (≥50), or inadequate (<50). Experimental protocols used for metrics calculations. Methods in solution: for (**1**), reference [18] was used; for (**2**), reference [36] was used. Mechanochemical methods: for (**1**), Table 1 entry 6; for (**2**), Table 2, entry 6.

The comparison of DOZN 3.0 scores for the preparation of (*S*)‐(‐)‐perillartine (**1**) and *N*‐*tert*‐butyl‐α‐phenylnitrone (PBN, **2**) is shown in Figure 2A (and in the Excel spreadsheet in the Supporting Information). For both compounds, the aggregate DOZN 3.0 scores favor the mechanochemical approach, indicating a lower overall environmental impact relative to the solution‐based methods. In the case of (*S*)‐(‐)‐perillartine (**1**), the scores for *Group 1* (Improved Resource Use) and *Group 3* (Reduced Human and Environmental Hazards) were significantly improved mainly due to the elimination of solvents for both the reaction and the recovery of the product, and for the simplified work‐up procedure.

Notably, analysis of the individual scores for *N*‐*tert*‐butyl‐α‐phenylnitrone (PBN, **2**) shows that the difference arises primarily from *Group 3* (Reduced Human and Environmental Hazards), mainly attributed to the use of dichloromethane and pyrrolidine in the solution‐based process [18], and not to a difference in yields (97% vs. 65% for solution vs. mechanochemistry), or reagents stoichiometry, used in equimolar amounts in both methods. For both compounds and synthetic methods (mechanochemical or solution), *Group 2* (Increased Energy Efficiency) does not contribute significantly to the final score, as all reactions were performed at room temperature and at atmospheric pressure, with only the use of reduced pressure in filtration processes and solvent evaporation (for the solution‐based process). It is important to note that the *Group 2* (Increased Energy Efficiency) scores for the mechanochemical protocols may be artificially favorable in the DOZN 3.0 assessment. This is due to the software's assumption that reactions performed at room temperature consume minimal energy, without accounting for the actual electrical consumption of the milling equipment. This limitation should be considered when interpreting DOZN 3.0 outputs for mechanochemical processes, especially in the context of a fair comparison with solution‐based methods.

For both compounds, aggregate scores for the mechanochemical methods (2.5 and 3.4, respectively, for **1** and **2**) were lower than the aggregate scores for the corresponding solution‐based syntheses (18.7 and 6.9, respectively). In the case of (*S*)‐(‐)‐perillartine (**1**), the aggregate score for the mechanochemical method was *ca.* 7.5 times lower than for solution‐based synthesis.

#### 2.1.3 Assessment using EcoScale toolkit [46]

The EcoScale toolkit is a semi‐quantitative assessment method designed to evaluate the efficiency and sustainability of synthetic routes by integrating multiple factors, including yield, cost, safety, technical setup, and environmental impact [46] (Figure 2B, Figure S4 and Excel spreadsheet in the Supporting Information). Each process starts with an initial ideal score of 100, from which penalty points are subtracted based on specific drawbacks such as hazardous reagents, excessive solvent use, high energy consumption, or complex purification steps. The final score classifies the process into one of three categories: excellent (≥75), acceptable (≥50), or inadequate (<50).

For the solution‐based synthesis of (*S*)‐(‐)‐perillartine (**1**) [18], the main score (41) is primarily the result of the lower yield (58%) and greater safety hazards linked to the use of cyclohexane and ethyl acetate during the work‐up procedure. In contrast, the mechanochemical process (using POM/ZrO_2_ milling media, Table 1 entry 6) avoided the use of harmful chemicals or organic solvents also for the recovery of the product, achieving a score of (75), the only penalty being safety hazards associated with the unavoidable use of hydroxylamine hydrochloride, which is indeed the reagent. Therefore, also in this case, the mechanochemical method outperformed compared to the solution‐based one.

In the case of *N*‐*tert*‐butyl‐α‐phenylnitrone (PBN, **2**), similar scores are obtained for both solution (65 score) and mechanochemical methods (69 score, using POM/ZrO_2_ milling media, Table 2 entry 6), although differences arise from different sources. For the solution process, the main penalty involved safety concerns due to the use of dichloromethane, ethyl acetate, and silica gel used for the recovery of the product, whereas a lower yield accounts for the main drawback in the mechanochemical process. Interestingly, despite the lower yield of the mechanochemical method (65%, Table 2 entry 6) in comparison with the solution synthesis (97% yield), the EcoScale values remain competitive, confirming once again the favorable eco‐footprint of mechanochemistry if the reaction and all the post‐reaction operation are well designed.

#### 2.1.4 Comparison between the green assessment tools: DOZN 3.0 vs. EcoScale

Depending on the tool chosen for metrics calculations and the reaction protocols selected, the assessment of the greenness of the processes can lead to different conclusions. Therefore, for the sake of comparisons, and to demonstrate that only one single tool is not sufficient to determine which process is the best from the point of view of the eco‐footprint, DOZN 3.0 and EcoScale were considered, both allowing to quantitatively evaluate also the safety and hazards (not scored in Chem21). The preparation of PBN (**2**) was used as benchmark and three methods were compared: in solution (97% yield) [36], and by ball milling, using as milling media SS (Method A, 100% yield) [38], or the POM/ZrO_2_ combination (Method B, 65% yield, Table 2, entry 6) (Figure S4 in the Supporting Information).

As a result, based on DOZN 3.0 output (Figure S4), Method B resulted in the best with the lowest aggregate score (3.4), followed by the synthesis in solution [36], having a much better score (6.9) than the mechanochemical method A [38] (scoring 22.2). The high aggregate score for the mechanochemical Method A [38] is a consequence of the postreaction operation to recover the final product by precipitation/filtration of the salts in dichloromethane, impacting on both *Group 1* (Improved Resource Use) and *Group 3* (assessing waste generation, its severity, and the hazard profile of the final product) scores. In particular, very high scores were obtained for the green chemistry principle #1 (Prevention, in *Group 1* — scoring 100) and principle #3 (Safety, in *Group 3* — scoring *ca.* 63).

However, the assessment is completely reversed when EcoScale is used (Figure S4), with the mechanochemical Method A [38] being the greenest, followed by Method B and then solution synthesis [36]. The assessment is mainly driven by the yield of the reaction. No penalty points are given if the reaction is quantitative, but the penalty points increase dramatically as soon as the yield decreases. Therefore, in the case of Method A [38] (100% yield), there was no penalty, and the mechanochemical method falls in the “excellent zone” (score 88, Figure S4). For Method B (Table 2, entry 6), a 65% yield accounted for −17 penalty points (with the maximum value being −50 points). Consequently, Method B was classified in the “acceptable” zone with an EcoScale score of 69. Whether the mechanochemical process selected for the calculations delivered higher yield, the EcoScale score increased. For example, for the process conducted in ZrO_2_ (Table 2, entry 3), a yield of 76% accounted for a five‐point penalty gain, bringing the mechanochemical process again in the “excellent” zone. However, the EcoScale fails to consider the amount of solvent used (organic or not) and the waste generated, and it does not give the same importance or weight as DOZN 3.0 for the environmental, safety, and societal implications, which make its use not so common for greenness assessment, as it can be seen in the case of Method A, when comparing Ecoscale and DOZN 3.0 scores.

In summary, all assessments presented herein converge on a common conclusion: mechanochemistry offers an efficient approach to synthesize (*S*)‐(‐)‐perillartine (**1**) and *N*‐*tert*‐butyl‐α‐phenylnitrone (PBN, **2**), particularly by simplifying reaction setup and work‐up procedures and eliminating the need for hazardous reagents and solvents, with better green chemistry metrics in comparison with the solution‐based methods.

## 3 Conclusion

The unprecedented, multigramscale mechanochemical preparation of (*S*)‐(‐)‐perillartine (**1**), a potent sweetener, and *N*‐*tert*‐butyl‐α‐phenylnitrone (PBN, **2**) a well‐known free‐radical spin trap that exhibits antioxidant, neuroprotective, and anti‐aging properties were described, and the influence of different mechanochemical processing conditions was studied. The use of “softer” polymeric jars (PTFE or POM) was considered as valid alternative to “harder” milling materials (SS or ZrO_2_). The use of POM milling media is, to the best of our knowledge, still underexplored in the field of mechanochemistry, and this work highlights its benefits compared to more traditional jars. In some cases, it demonstrated better performance than PTFE, positioning it as a valuable and potentially more sustainable alternative at reduced cost. This claim is based on the broader context of avoiding fluoropolymers, given the growing environmental and health concerns surrounding per‐ and polyfluoroalkyl substances (PFAS), progressively identified as persistent environmental pollutants, the chemical class to which PTFE belongs. By offering a fluorine‐free alternative, POM aligns with a proactive “Safe and Sustainable by Design” strategy, mitigating future risks associated with persistent pollutants. Moreover, POM is also a valid alternative to SS, preventing or diminishing the probability of residual metal impurities due to abrasion of the milling media. Although, at present, POM jars are not commercially available, proper scale‐up would require the manufacture of larger POM jars (for batch) or POM‐coated screws (for continuous processes such as twin‐screw extrusion). When it comes to the use of planetary ball‐mills, the jars should be designed and manufactured to resist at the high energetic conditions provided by planetary ball‐mills, usually not compatible with “soft” polymeric materials (e.g., PTFE, PMMA, POM), which would break or deform during the milling process. These engineering aspects, however, lie beyond the scope of the current manuscript. By analogy with PTFE, which has already led to marketed large‐scale solutions, we believe that the present study on POM provides the necessary foundation to serve as a reference for future developments.

The comparative assessment of mechanochemical and solution‐based methods for the synthesis of (*S*)‐(‐)‐perillartine (**1**) and *N*‐*tert*‐butyl‐α‐phenylnitrone (PBN, **2**) highlights the environmental benefits of mechanochemistry. The comprehensive sustainability assessment performed by a comparative green evaluation using multiple complementary tools (Chem21, DOZN 3.0, and EcoScale) covering the entire process, including the downstream work‐up, showed that mechanochemical approaches consistently led to reduced solvent use, simplified work‐up procedures, and lower associated environmental and safety risks. This in‐depth analysis of the processes’ full eco‐footprint is a key contribution that moves beyond simple reaction yields.

Although some individual parameters, such as yield or atom economy, were initially more favorable in the solution‐based protocols, the overall performance of the mechanochemical methods demonstrated a lower environmental impact and greater alignment with green chemistry principles. In addition, the byproducts and waste generated by these methods exhibit minimal or no toxicity to humans and the environment. Ball‐milling conditions differ significantly from conventional experimental setups [77], and as such, we could not assume that the *E*,*Z*‐isomeric ratio would mirror that observed under standard conditions in solution. Through comparison with literature spectral data, we determined that the products synthesized under ball‐milling conditions were consistent with those previously reported and with full selectivity toward the regioisomers *E*‐(**1**) [21] and the more stable form *Z* [78] for PBN (**2**).

This work contributes to the field by bridging practical synthesis with detailed sustainability analysis. Specifically, its novelty lies in (i) applying mechanochemistry to synthesize high‐value, marketable ingredients (e.g., perillartine) for the food and personal care sectors [79] from an abundant, renewable biomass‐derived aldehyde (e.g*.*, perillaldehyde) [80], using fluorine‐free polymeric jars; (ii) demonstrating the process's robustness and versatility across different, commonly used milling platforms; (iii) closely working with manufacturers for mechanochemical devices (for both batch and continuous processes) to design new reactors, or to retool and retrofit existing devices for new applications, at different scales; and (iv) providing a comprehensive green assessment of the entire workflow, including the often‐neglected downstream processing, which validates the method's alignment with SSbD principles and the IUPAC Guiding Principles of Responsible chemistry [11].

We hope that this contribution will stimulate the curiosity of the scientific community and contribute to a circular economy mindset, by transforming a readily available natural raw material from *Perilla frutescens* into a commercially relevant product. Our study bridges the gap between green chemistry metrics and industrial application, and it demonstrates that mechanochemical protocols are not only sustainable and efficient but can also deliver products of tangible economic significance, thereby linking practical synthesis with sustainability principles. The cross‐fertilization among several market sectors raises awareness on innovation opportunities for a more sustainable chemical industry, which should also result in new developments in mechanochemistry. Mechanochemical routes can become synthetically superior to the current manufacturing processes in solution, upon further developments at process‐level.

## Supporting Information

Additional supporting information can be found online in the Supporting Information section. The authors have cited additional references within the Supporting Information [81]. The data supporting this article, including ^1^H and ^13^C NMR, FT‐IR (ATR) spectra for compounds (**1**) and (**2**), excel spreadsheets for green chemistry metrics calculations by Chem21, DOZN tools and EcoScale data input are available as part of Supplementaty Information.

## Author Contributions

**Evelina Colacino**: conceptualization, project administration, data curation, funding acquisition, supervision, resources, writing, review and editing. **Nicolas Fantozzi**: investigation, formal analysis, writing – original draft. **Rubén Solórzano‐Rodríguez**: conceptualization, data curation, formal analysis, writing – original and final draft, supervision, visualization, review and editing. **Andrea Casagrande**: investigation, formal analysis, validation. **Christos M. Chatzigiannis** and **Corentin Bordier**: visualization, review and editing. **Pietro Rando**: review and editing, funding.

## Funding

This study was supported by European Cooperation in Science and Technology (CA18112), Région Occitanie Pyrénées‐Méditerranée (Pre‐Maturation 2020 – MECHAPI grant (ESRPREMAT – 00262), European Health and Digital Executive Agency (101057286), Campus France ‐ French Embassy in Estonia.

## Conflicts of Interest

The authors declare no conflicts of interest.

## Supporting information

Supplementary Material

## Data Availability

The data that support the findings of this study are available in the supplementary material of this article.
