# Supplementary material for: Safe and Sustainable by Design (SSbD) Synthesis of Food and Personal Care Ingredients by Mechanochemistry
Source: ChemSusChem. 2026 May 4;19(9):e202502491. doi: 10.1002/cssc.202502491 (PMC13139749; doi:10.1002/cssc.202502491)
Supplement: Supplementary file 1 — Supplementary Material [file CSSC-19-e202502491-s001.zip › cssc70628-sup-0001-SuppData-S1.pdf]

SUPPORTING INFORMATION

Safe and Sustainable by Design (SSbD) synthesis of food and personal care ingredients by mechanochemistry.

Rubén Solórzano-Rodríguez,[a,†] Nicolas Fantozzi,[a,†] Andrea Casagrande,[a,b] Christos M. Chatzigiannis,[a] Corentin Bordier,[a] Pietro Rando,[c] and Evelina Colacino*[a]

[a]ICGM, Univ Montpellier, CNRS, ENSCM, 34293 Montpellier, France, *E-mail: evelina.colacino@umontpellier.fr

[b] Tallin University of Technology, Järveküla tee 75, 30322, Ida-Virumaa, Estonia

[c] CRB Benelux BV, Oxfordlaan 70, 6229 EV Maastricht, The Netherlands

† Equal contribution

INDEX

Experimental part – General remarks and experimental procedures	S3
Experimental procedures	S4
**Table S1**. Weight for the grinding balls used for the experiments.	S4
**Figure S1.** In-house made polyoxymethylene (POM) jars (nominal volume: 10 mL).	S5
General procedure for the synthesis of (*S,E*)-(-)-perillartine (1) in a vibrating ball-mill.	S5
General procedure for the synthesis of (*S,E*)-(-)-perillartine (1) in a planetary ball-mill	S6
**Figure S2.** ATR FT-IR spectrum of (*S,E*)-(-)-perillartine (**1**) prepared by ball-milling (Table 1, entry 6).	S7
General procedure for the synthesis of *N*-*tert*-butyl-α-phenylnitrone (PBN, **2**) in a vibrating ball-mill.	S7
General procedure for the synthesis of *N*-*tert*-butyl-α-phenylnitrone (PBN, **2**) in a planetary ball-mill.	S7
**Figure S3.** ATR FT-IR spectrum of *N*-*tert*-butyl-α-phenylnitrone (PBN, **2**) prepared by ball-milling (Table 2, entry 6).	S8
**Table S2.** Electrical energy consumption associated with milling experiments reported in Tables 1 and 2.	S9
**Green chemistry metrics**	S9
**Figure S4.** Comparison of green chemistry metrics calculators for PBN (**2**)	S9
**References**	S10
**Figure S5.** ^{1}H NMR spectrum of (*S,E*)-(-)-perillartine (**1**) in $CDCl_3$	S11
**Figure S6.** ^{1}H NMR spectrum of (*S,E*)-(-)-perillartine (**1**) in DMSO-d_6	S12
**Figure S7.** ^{13}C NMR spectrum of (*S,E*)-(-)-perillartine (**1**) in DMSO-d_6	S13
**Figure S8.** ^{1}H NMR spectrum of *N*-*tert*-butyl-α-phenylnitrone (PBN, **2**) in $CDCl_3$	S14
**Figure S9.** ^{1}H NMR spectrum of *N*-*tert*-butyl-α-phenylnitrone (PBN, **2**) in DMSO-d_6	S15
**Figure S10.** ^{13}C NMR spectrum of of *N*-*tert*-butyl-α-phenylnitrone (PBN, **2**) in DMSO-d_6	S16

EXPERIMENTAL PART

General remarks and experimental procedures

MATERIALS AND METHODS

Chemicals. All reagents were commercially available and used without any further purification.

Liquid nuclear magnetic resonance (NMR) spectroscopy. ^{1}H and ^{13}C NMR spectra were recorded on a Bruker 400 MHz spectrometer at room temperature in DMSO-d_6. Chemical shifts (δ) of ^{1}H NMR and ^{13}C NMR spectra are reported in ppm relative to residual solvent signals (DMSO in DMSO-d$_6$: δ = 2.50 ppm for ^{1}H and δ = 39.52 ppm for ^{13}C NMR); *J* values are given in *Hz*. The multiplicity was reported with the following abbreviations: s = singlet, d = doublet, t = triplet, q = quartet, m = multiplet, br = broad signal peak.

Melting point. For melting point in a capillary measurement, an automatic Stuart SMP50 was utilized equipped with a PT1000 Platinum resistance temperature sensor and a heating rate of 5°C/min.

Fourier-transform infrared spectroscopy (FT-IR). Fourier-transform infrared spectra were recorded in the ATR mode on a Perkin Elmer Spectrum 100 FT-IR instrument (Attenuated Total Reflectance Infrared ATR-IR device) in reflectance mode. The characteristic IR absorptions mentioned in the text are reported in cm^{-1}. Data are reported according to their group absorption regions as s = strong, m = medium, and w = weak.

Liquid Chromatography coupled to Mass Spectrometry (LC-MS).
LC-MS measurements were performed on a UPLC Ultimate 3000 (Thermo) with a Kinetex EVO C18 100A 50 x 2.1, 1.7µm (Phenomenex) column, flow 0.5 mL/min linear gradient CH_3CN in water 0-100% (+ 0.1% HCO_2H) in 3 min.

Gas Chromatography coupled to Mass Spectrometry (GC-MS). GC-MS analysis was performed using a Shimadzu GCMS-QP2010 SE (Shimadzu, Kyoto, Japan) gas chromatograph, equipped with an AOC-20i autosampler and a Zebron ZB-5ms column equipped and a data processor. The oven program was as follows: temperature increase from 50 °C at a rate of 2°C/min up to 280 °C. Helium was used as carrier gas (0.74 mL min^{-1}). The injector and detector temperatures were set at 250 °C and 280 °C, respectively. 1 µL of the solution was injected with split mode 30; MS range 30-800.

HRMS analyses were performed a Q-TOF mass analyzer (Waters, 2001) with ESI ionization mode [Tolerance = 1.0 mDa / DBE: min = -3.0, max = 100.0 , Element prediction: Off, Number of isotope peaks used for i-FIT = 3, Monoisotopic Mass, Even Electron Ions 1979 formula(e) evaluated with 4 results within limits (up to 50 best isotopic matches for each mass).

Ball-milling experiments were performed in a:

a) MM400 horizontal vibrating ball mill (Retsch GmbH, Haan, Germany) – Table 1 (entries 1-9) and Table 2 (entries 1-6), at 30 *Hz* – Jar volume: 10 mL; Materials: Stainless Steel (SS), Zirconium Oxide (ZrO_2), polytetrafluoroethylene (PTFE) and in-house made polyoxymethylene (POM) (*c.f.*, Figure S1);
b) Pulverisette P23 vertical vibrating ball mill (Fritsch, Idar-Oberstein, Germany) - Table 1 (entries 1-2) and Table 2 (entries 7-9), at 50 *Hz* – Jar volume (material) : 5 mL (PTFE), 10 mL (SS);
c) Pulverisette P7 Classic (Fritsch, Idar-Oberstein, Germany) – Table 1 (entries 13 and 14) and Table 2 (entries 10 and 11), at 450 rpm – Jar Volume (material) : 12 mL (SS, ZrO_2);
d) PM100 planetary ball-mill (Retsch GmbH, Haan, Germany) – Table 1 (entries 15 and 16), at 450 rpm – Jar volume (material) : 45 mL (ZrO_2).

Table S1. Weight for the grinding balls used for the experiments.

	**Weight for each ball (g)**						
	RETSCH			FRITSCH			
Balls diameter (Ø) in mm	**5**	**7**	**10**	**3**	**5**	**7**	**10**
Stainless steel (SS)	0.506	1.388	4.010	0.108	0.500	n.a.[a]	4.035
Zirconium oxide (ZrO_2)	0.414	1.093	3.392	0.088	0.384	0.829	2.948

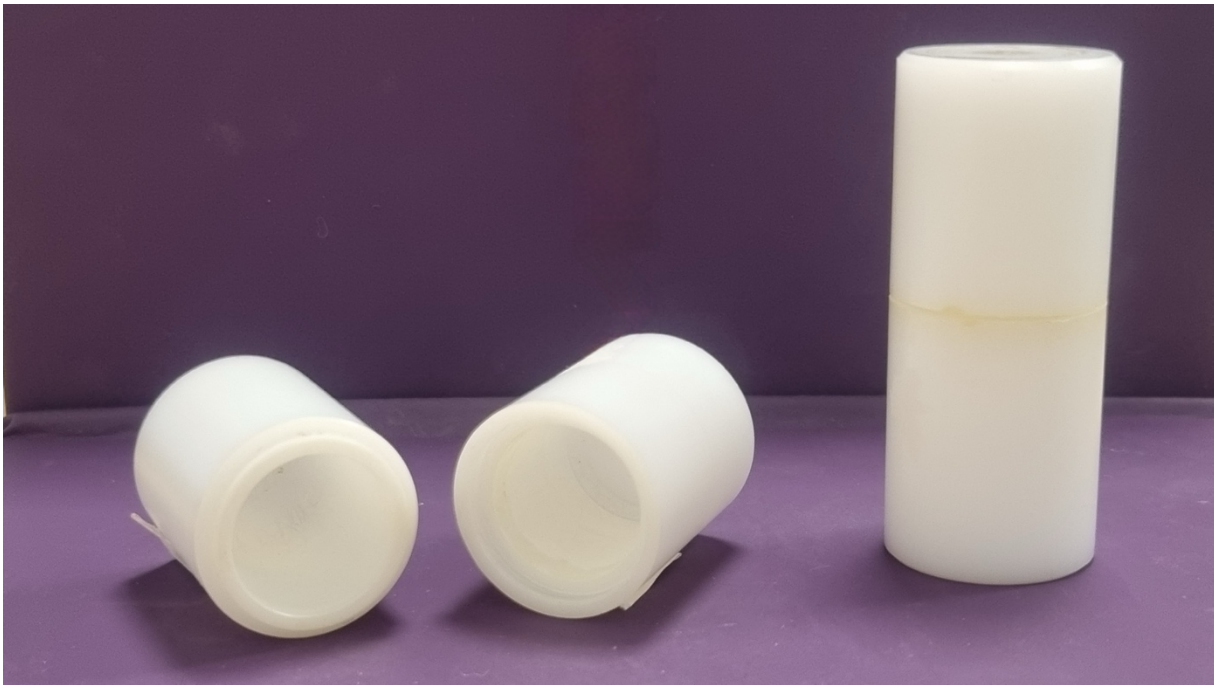

Figure S1. In-house made polyoxymethylene (POM) jars (nominal volume: 10 mL).

EXPERIMENTAL PROCEDURES

General procedure for the synthesis of (*S,E*)-(-)-perillartine (1) in a vibrating ball-mill (Table 1, entries 1-12). CAS [319907-28-3]**.** A mixture of (*S*)-(-)-perillaldehyde (4.0 mmol, 1.0 equiv.), hydroxylamine hydrochloride (4.0 mmol, 1.0 equiv.), and $NaHCO_3$ or K_2HPO_4 (4 mmol, 1.0 equiv.) was introduced into a 5 mL (entries 1-10) or 10 mL (entries 8-9) jar containing milling balls, as per the conditions specified in Table 1. The reactants were milled at 30 *Hz* (or 50 *Hz*) for 60 min. Aqueous $NaHSO_3$ (10% w/w) (1.5 mL for 5 mL, or 2.5 mL for the 10 mL jar) was then added to the jar and the crude mixture was milled at 25 *Hz* for 5 min. The resulting precipitate was isolated by filtration, washing with water (10 mL). After drying over P_2O_5, *in vacuo* overnight, (*S*)-(-)-perillartine (1) was obtained as a white solid (*E*-regioisomer only, determined by comparison with the NMR spectrum recorded in $CDCl_3$ available in the literature[1]). For entry 1: 99% yield (654.3 mg); for entry 6: 98% yield (647.7 mg).

General procedure for the synthesis of (*S,E*)-(-)-perillartine (1) in a planetary ball-mill (Table 1, entries 13-16). CAS [319907-28-3]**.** A mixture of (*S*)-(-)-perillaldehyde (4.0 or 30.0 mmol, 1.0 equiv.), hydroxylamine hydrochloride (4.0 or 30.0 mmol, 1.0 equiv.), and $NaHCO_3$ or K_2HPO_4 (4.0 or 30.0 mmol, 1.0 equiv.) was introduced into a 12 mL (entries 13 and 14) or 50 mL (entries 15 and 16) jar containing milling balls, as per the conditions specified in Table 1. The reactants were milled at 450 rpm for 60 min. Aqueous $NaHSO_3$ (10% w/w) (2.5 mL for 12 mL jar, or 15 mL for 50 mL) was then added to the jar and the crude mixture was milled at 300 rpm for 5 min. The resulting precipitate was isolated by filtration washing with water (10 mL-20 mL). After drying over P_2O_5, *in vacuo* overnight, (*S*)-(-)-perillartine (1) was obtained as a white solid (*E*-regioisomer only, determined by comparison with the NMR spectrum recorded in $CDCl_3$ available in the literature[1]). For entry 13: 98% yield (647.7 mg), For entry 15: 90% yield (4.46 g).

^{1}H NMR (400 M*Hz*, $CDCl_3$)[1] δ (ppm): 7.71 (s, C=NO, 1H), 6.08 – 6.03 (m, 1H), 4.76 – 4.70 (*pseudo*-d, 2H), 2.46 -2.38 (m, 1H), 2.36 – 2.27 (m, 1H), 2.26 -2.05 (m, 3H), 1.93 – 1.84 (m, 1H), 1,74 (s, 3H), 1.54 – 1.43 (m, 1H). **^{1}H NMR** (400 M*Hz*, DMSO-d_6) δ (ppm): 10.73 (s, C=NO, 1H), 7.67 (s, 1H), 6.03 – 5.98 (m, 1H), 4.73 -4.70 (m, 2H), 2.41 – 2.31

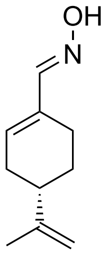

(**1**)

(m, 1H), 2.40 – 2.31 (m, 1H), 2.29 – 2.20 (m, 1H), 2.21 – 1.97 (m, 3H), 1.83 – 1.77 (m, 1H), 1.71 (s, 3H), 1.45 – 1.35 (m, 1H); **^{13}C NMR** (101 M*Hz*, DMSO-d_6) δ (ppm): 150.7, 148.9, 132.8, 132.6, 109.1, 40.3, 30.5, 26.5, 23.7, 20.6; **HRMS ESI-(+)** calcd for $C_{10}H_{16}NO$ $[M+H]^+$ 166.1232, found 166.1236. **m.p.** (*E*-regioisomer) 90.2-93.5°C (lit. 102°C);[2] **FT-IR (ATR)** ν_{max}/cm^{-1} : 3249 (m_{broad},νO-H), 2931 (m, νC=CH), 1642 (s, νC=N), 1454 (m), 1434 (m), 1422 (m), 1291 (m), 1240 (w), 1174 (m), 1147 (w), 1011 (w), 985 (m), 953 (s), 934 (s), 906 (s), 891 (s), 803 (m), 704 (s).

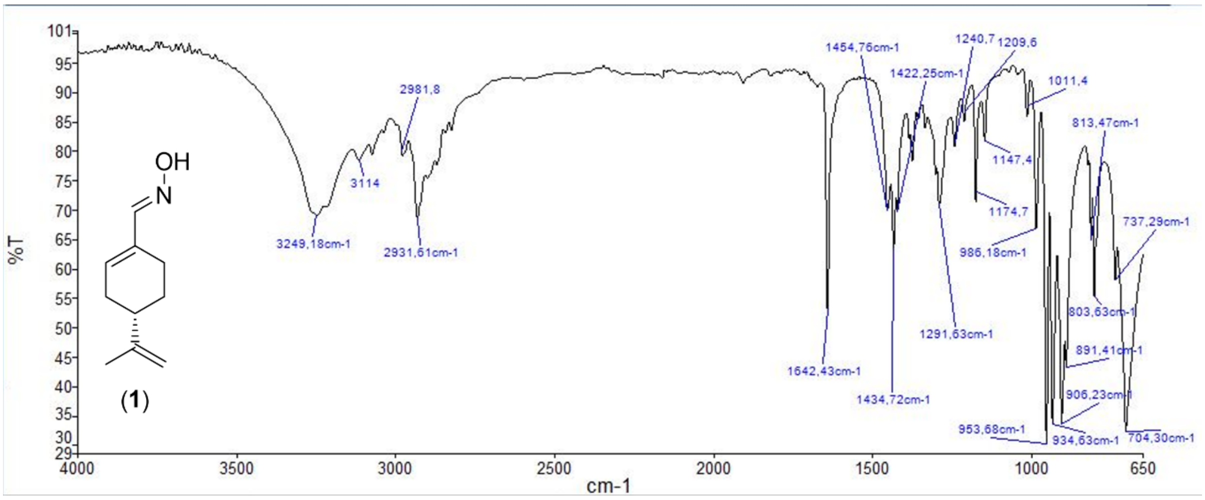

Figure S2. ATR FT-IR spectrum of (*S,E*)-(-)-perillartine (**1**) prepared by ball-milling (Table 1, entry 6).

General procedure for the synthesis of *N-tert*-butyl-α-phenylnitrone (PBN, 2) in a vibrating ball-mill (Table 2, entries 2-9)**.** CAS [3376-24-7]**.** A mixture of (4.0 mmol), *N-tert*-butyl hydroxylamine hydrochloride (4.0 mmol, 1.0 equiv.), and $NaHCO_3$ (4.0 mmol, 1.0 equiv.) was introduced into a 5 mL (entries 8-9) or 10 mL (entries 2-7) jar containing milling balls, as per the conditions specified in Table 2. The reactants were milled at 30 *Hz* (or 50 *Hz*) for 60-120 min. Aqueous $NaHSO_3$ (10% w/w) (2 mL for 5 mL, or 2.5 mL for the 10 mL jar) was then added to the jar and the crude mixture was milled at 25 *Hz* for 5 min. The resulting precipitate was isolated by filtration, washing with water (10 mL). After drying over P_2O_5, *in vacuo* overnight, *N-tert*-butyl-α-phenylnitrone (PBN, **2**) was obtained as a white solid (*Z*-regioisomer only, determined by comparison with the NMR

spectrum recorded in $CDCl_3$ available in the literature[3]). For entry 3: 76% yield (538.8 mg); for entry 6: 65% yield (460.8 mg).

General procedure for the synthesis of *N-tert*-butyl-α-phenylnitrone (PBN, 2) in a planetary ball-mill (Table 2, entries 10-11). CAS [3376-24-7]**.** A mixture of (4.0 mmol), *N-tert*-butyl hydroxylamine hydrochloride (4.0 mmol, 1.0 equiv.), and K_2HPO_4 (4.0 mmol, 1.0 equiv.) was introduced into a 12 mL jar containing milling balls, as per the conditions specified in Table 2. The reactants were milled at 450 rpm for 60 min. Aqueous $NaHSO_3$ (10% w/w) (5 mL) was then added to the jar and the crude mixture was milled at 300 rpm for 5 min. The resulting precipitate was isolated by filtration, washing with water (10 mL). After drying over P_2O_5, *in vacuo* overnight, *N-tert*-butyl-α-phenylnitrone (PBN, **2**) was obtained as a white solid (*Z*-regioisomer only, determined by comparison with the NMR spectrum recorded in $CDCl_3$ available in the literature[3]). For entry 10: 79% yield (560.1 mg).

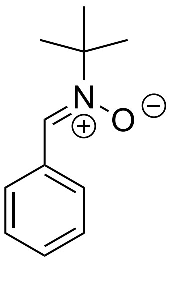

(**2**)

For the *Z*-regioisomer: **^{1}H NMR** (400 M*Hz*, $CDCl_3$)[3] *δ* (ppm): 8.31 - 8.25 (m, ArH, 2H), 7.55 (s, CH=NO, 1H), 7.44 - 7.37 (m, ArH, 3H), 1.61 (s, $C(CH_3)_3$, 9H); **^{1}H NMR** (400 M*Hz*, DMSO-d_6) *δ* (ppm): 8.37 – 8.34 (m, ArH, 2H), 7.84 (s, CH=NO, 1H), 7.44 – 7.36 (m, ArH, 3H), 1.50 (s, $C(CH_3)_3$, 9H); **^{13}C NMR** (101 M*Hz*, DMSO-d_6) *δ* (ppm): 131.6, 129.5, 128.6, 128.3, 128.1, 70.4, 27.9; **HRMS ESI-(+)** calcd for $C_{11}H_{16}NO$ $[M+H]^+$ 178.1232, found 178.1225. **m.p.** (*Z*-regioisomer) 70-72°C (by precipitation in water) (lit. 71-72°C, by recrystallisation in hexane,[4] or upon *in vacuo* evaporation from CH_2Cl_2)[3]; **FT-IR (ATR)** ν_{max}/cm^{-1} 3086 (w), 3059 (w), 2974 (νN-O), 1579 and 1567 (m, νC=N), 1454 (m), 1444 (m), 1408 (w), 1365 (s), 1320 (w), 1248 (w), 1192 (s), 1116 (s), 1077 (m), 1024 (m), 929 (w), 904 (m), 836 (m), 758 (s), 696 (s).

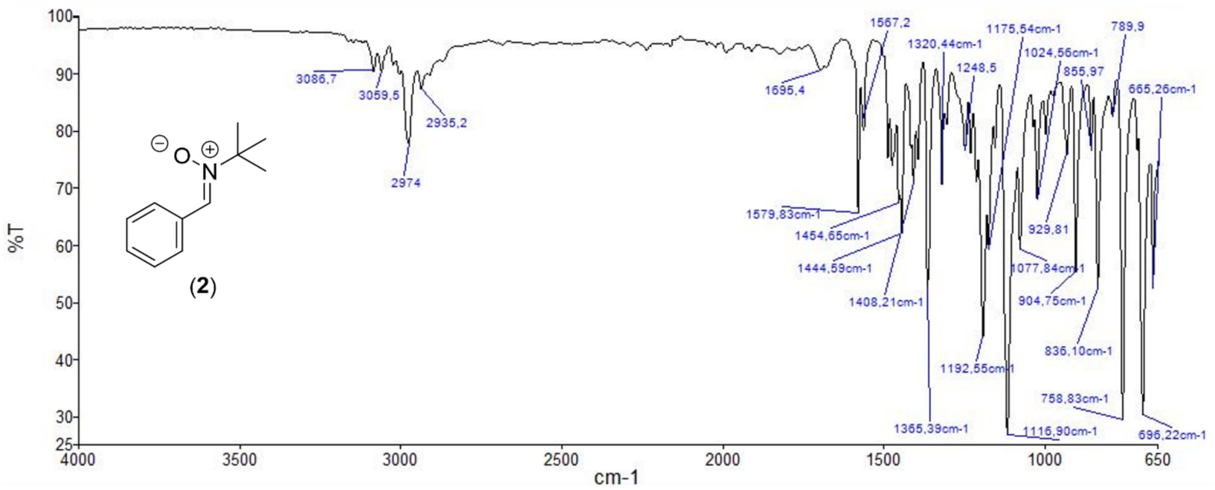

Figure S3. ATR FT-IR spectrum of *N-tert*-butyl-α-phenylnitrone (PBN, **2**) prepared by ball-milling (Table 2, entry 6).

Table S2. Electrical energy consumption associated with milling experiments reported in Tables 1 and 2 (measured with an Energy Monitor Plug Power Meter).

**Data from Table 1**				
Entry #	Type of ball-mill	Speed	Time (h)	Power (kWh)
1-6	Horizontal mixer-mill MM400	30 *Hz*	1	0.23
7-9	Horizontal mixer-mill MM400	30 *Hz*	1	0.13
10-12	Vertical mixer-mill P23	50 *Hz*	1	0.03
13, 14	Planetary mill P7 Classic line	450 rpm	1	0.13
15, 16	Planetary mill PM100	450 rpm	1	0.27
**Data from Table 2**				
Entry #	Type of ball-mill	Speed	Time (h)	Power (kWh)
2-6	Horizontal mixer-mill MM400	30 *Hz*	2	0.46
7-9	Vertical mixer-mill P23	50 *Hz*	1	0.03
10, 11	Planetary mill P7 Classic line	450 rpm	1	0.13

GREEN CHEMISTRY METRICS

The calculations of the green metrics using Chem21,[5] DOZN™ 3.0[6] and EcoScale[7] toolkits for the solution syntheses of compounds (*S,E*)-(-)-perillartine (**1**)[8] and *Z-N-tert*-butyl-α-phenylnitrone (PBN, **2**)[9] were performed based on the experimental

procedures reported in literature. The detailed calculations are also available as a separate excel files, as integral part of the ESI.

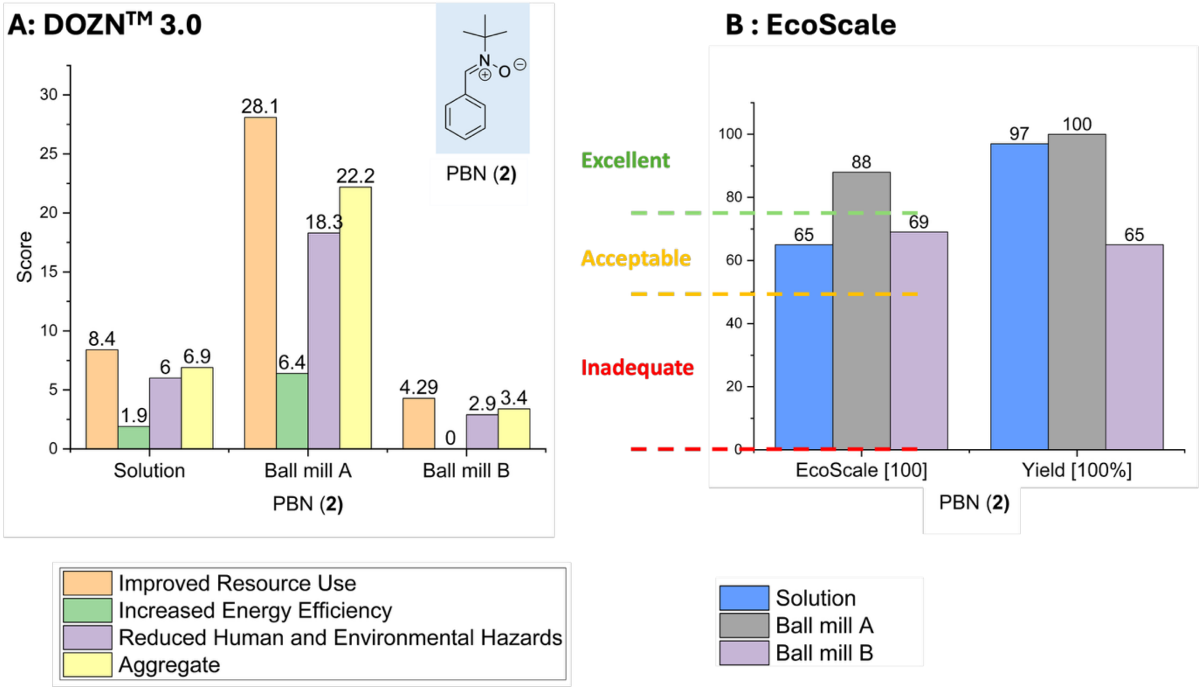

Figure S4. Comparison of green chemistry metrics calculators for PBN (**2**). A) DOZN™ 3.0; B) Ecoscale. *Legend* (for experimental protocols): Solution method: from reference 9 – 97% yield; Ball Mill A method: from reference 3 – 100% yield (work-up: precipitation by CH_2Cl_2/filtration); Ball mill B method: Table 2, entry 6 – 65% yield (work-up: aqueous precipitation/filtration).

REFERENCES

[1] P. Zambelli, A. Pinto, D. Romano, E. Crotti, P. Conti, L. Tamborini, R. Villa, F. Molinari, *Green Chem.* **2012**, *14*, 2158-2161.

[2] X.-Y. Yuan, Z. M., S. Weng Ng, *Acta Cryst.* **2009**, *E65*, o2149.

[3] E. Colacino, P. Nun, F. M. Colacino, J. Martinez, F. Lamaty, *Tetrahedron* **2008**, *64*, 5569-5576.

[4] M. Shimizu, I. Shibuya, Y. Taguchi, S. Hamakawa, K. Suzuki, T. Hayakawa, *J. Chem. Soc., Perkin Trans. 1* **1997**, 3491–3492.

[5] C. R. McElroy, A. Constantinou, L. C. Jones, L. Summerton, J. H. Clark, *Green Chem.* **2015**, *17*, 3111-3121.

[6] A. DeVierno Kreuder, T. House-Knight, J. Whitford, E. Ponnusamy, P. Miller, N. Jesse, R. Rodenborn, S. Sayag, M. Gebel, I. Aped, I. Sharfstein, E. Manaster, I. Ergaz, A. Harris, L. Nelowet Grice, *ACS Sustainable Chem. Eng.* **2017**, *5*, 2927-2935. DOZN™ is a universal tool, suitable for scoring any process, no matter the activation technique used, once the detailed experimental/process conditions are known. The DOZN tool is

accessible free of charge here: https://bioinfo.merckgroup.com/dozn (accessed June 27, 2025).
[7] K. Van Aken, L. Strekowski, L. Patiny, *Beilstein J. Org. Chem.* **2006**, *2*. The EcoScale tool is accessible free of charge here: https://ecoscale.cheminfo.org (accessed 11 June, 2025).
[8] T. Betke, P. Rommelmann, K. Oike, Y. Asano, H. Gröger, *Angew. Chem. Int. Ed.* **2017**, *56*, 12361-12366.
[9] S. Morales, F. G. Guijarro, I. Alonso, J. L. García Ruano, M. B. Cid, *ACS Catal.* **2016**, *6*, 84-91.

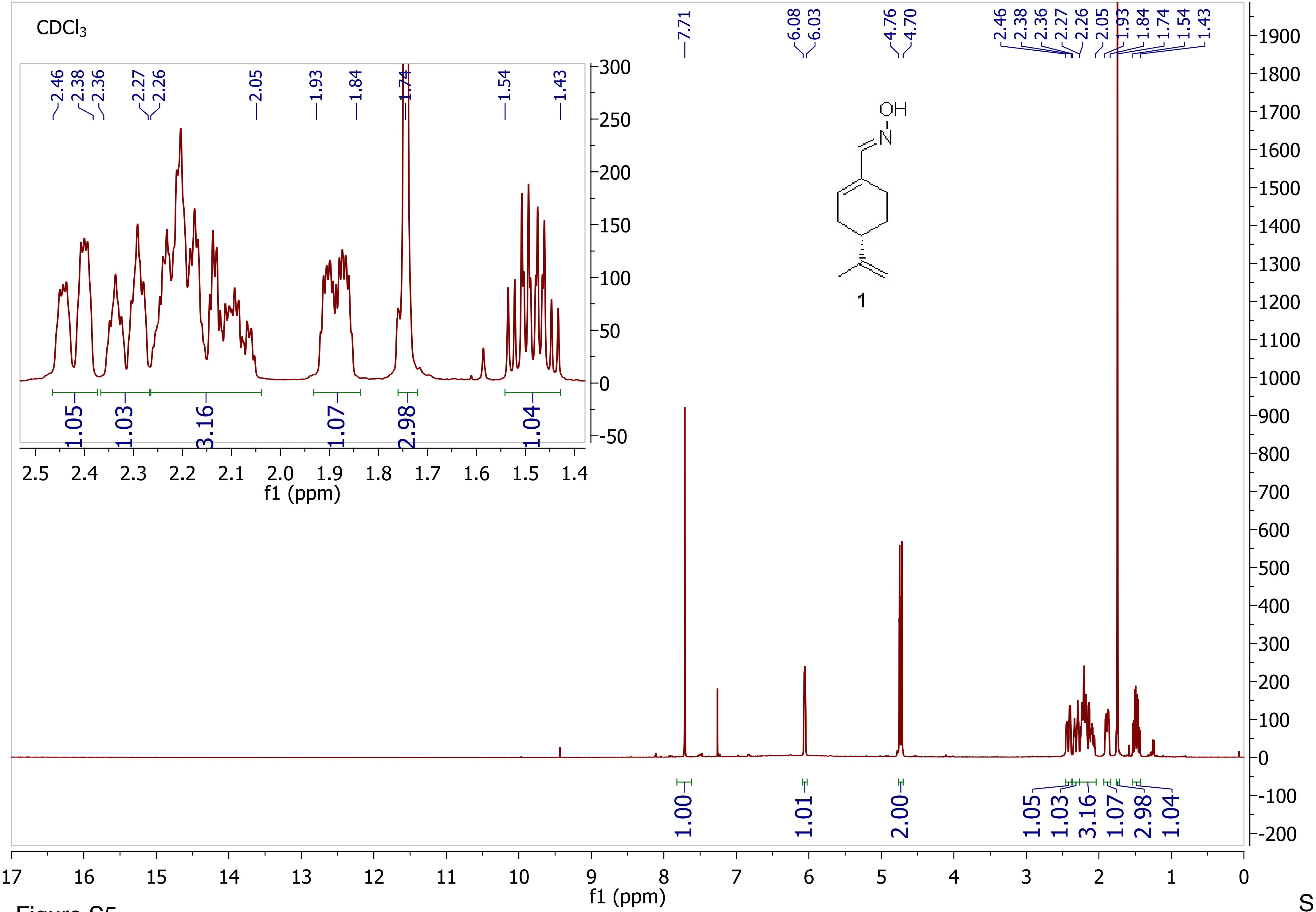

Figure S5

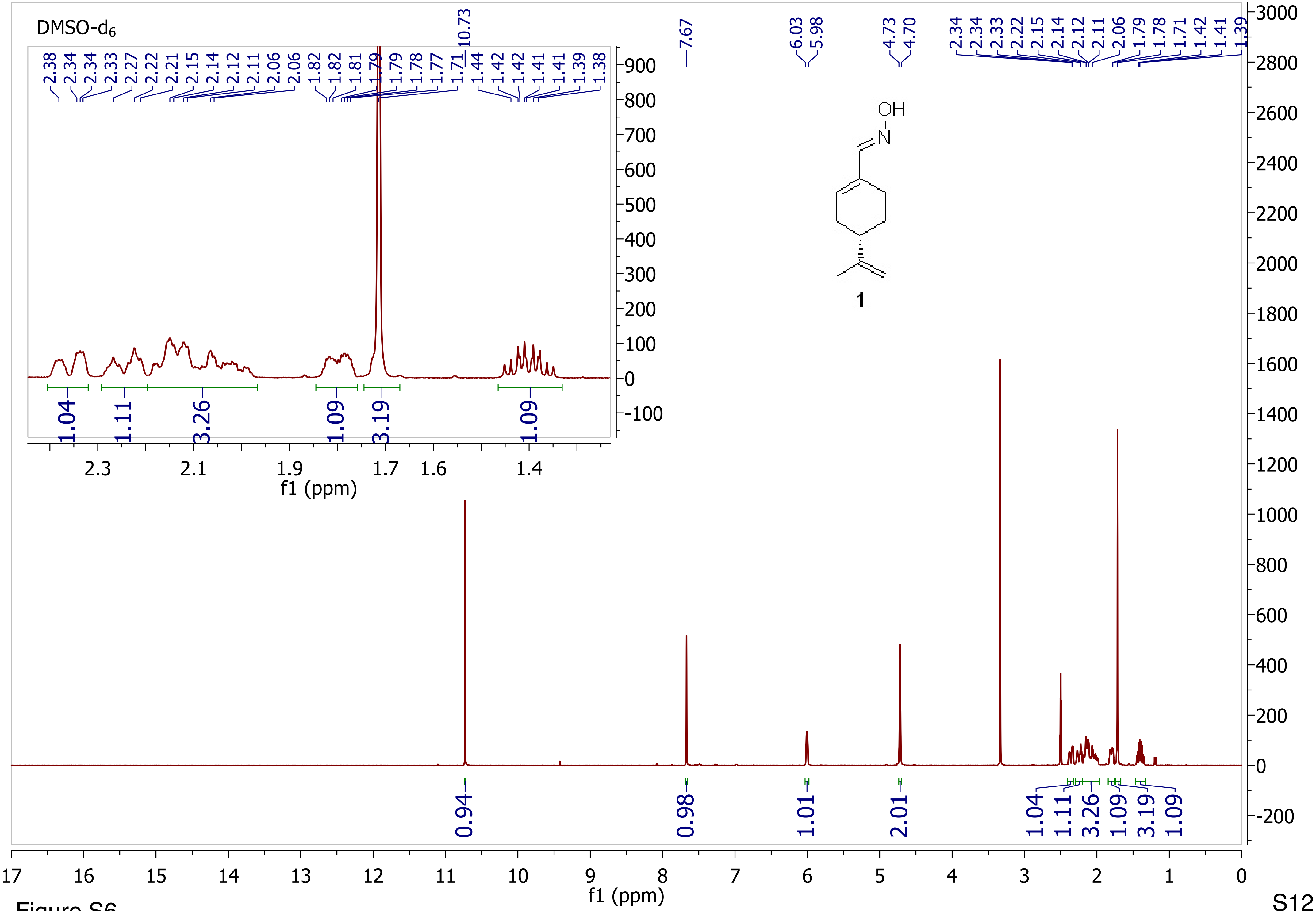

Figure S6

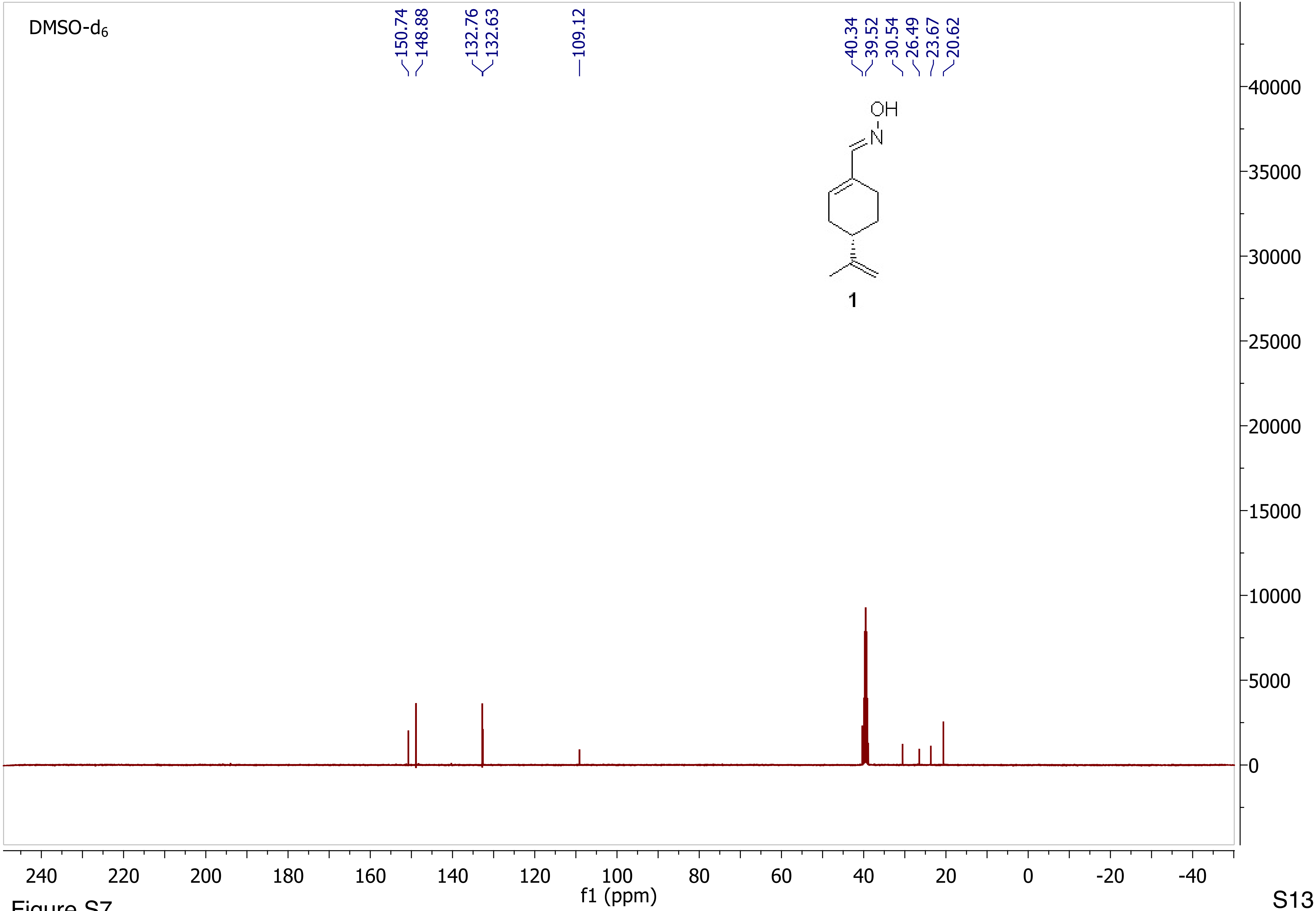
DMSO-d6
150.74
148.88
132.76
132.63
109.12
40.34
39.52
30.54
26.49
23.67
20.62
OH
N
1
40000
35000
30000
25000
20000
15000
10000
5000
0
240
220
200
180
160
140
120
100
80
60
40
20
0
-20
-40
f1 (ppm)

Figure S7

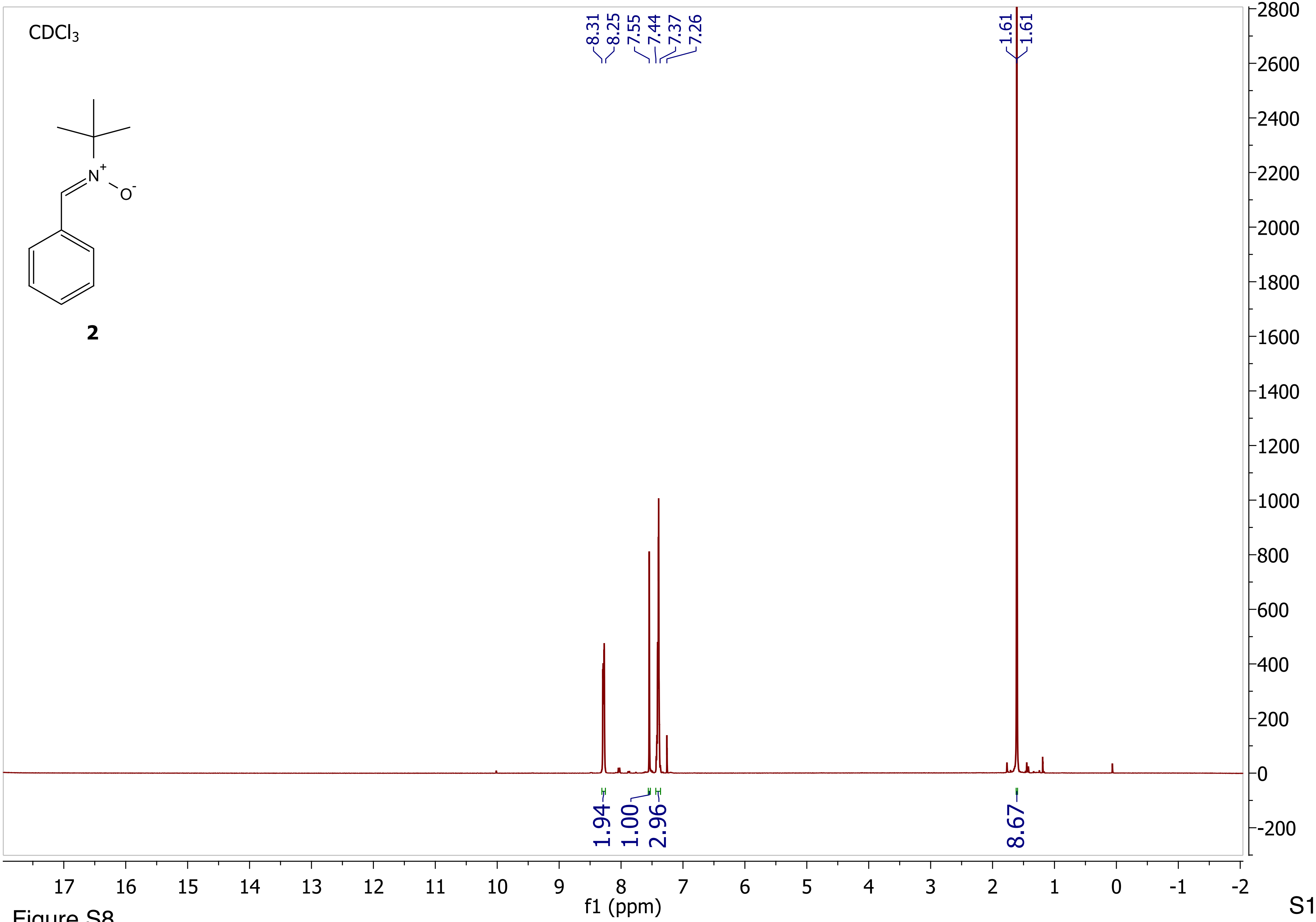

Figure S8

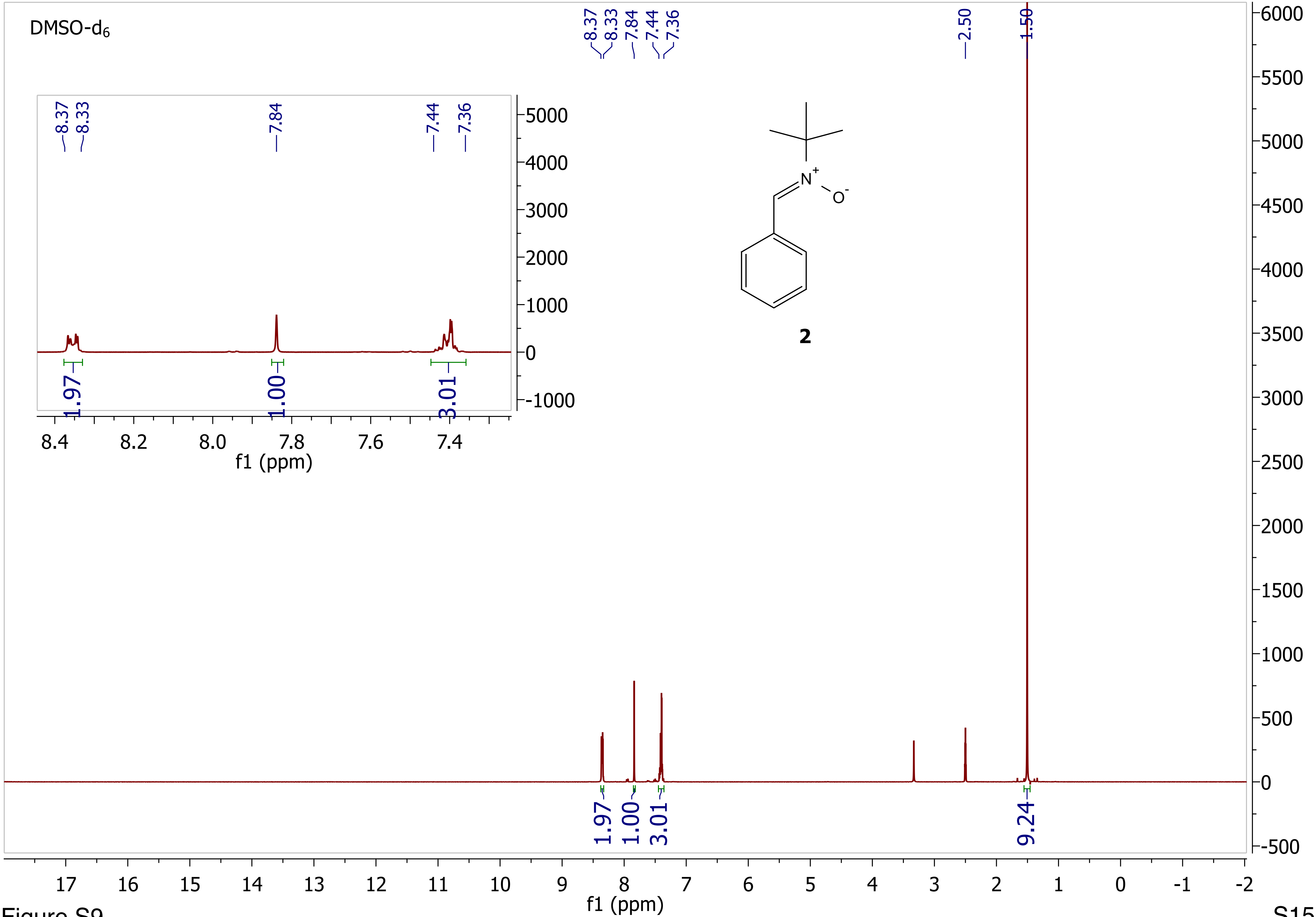

Figure S9

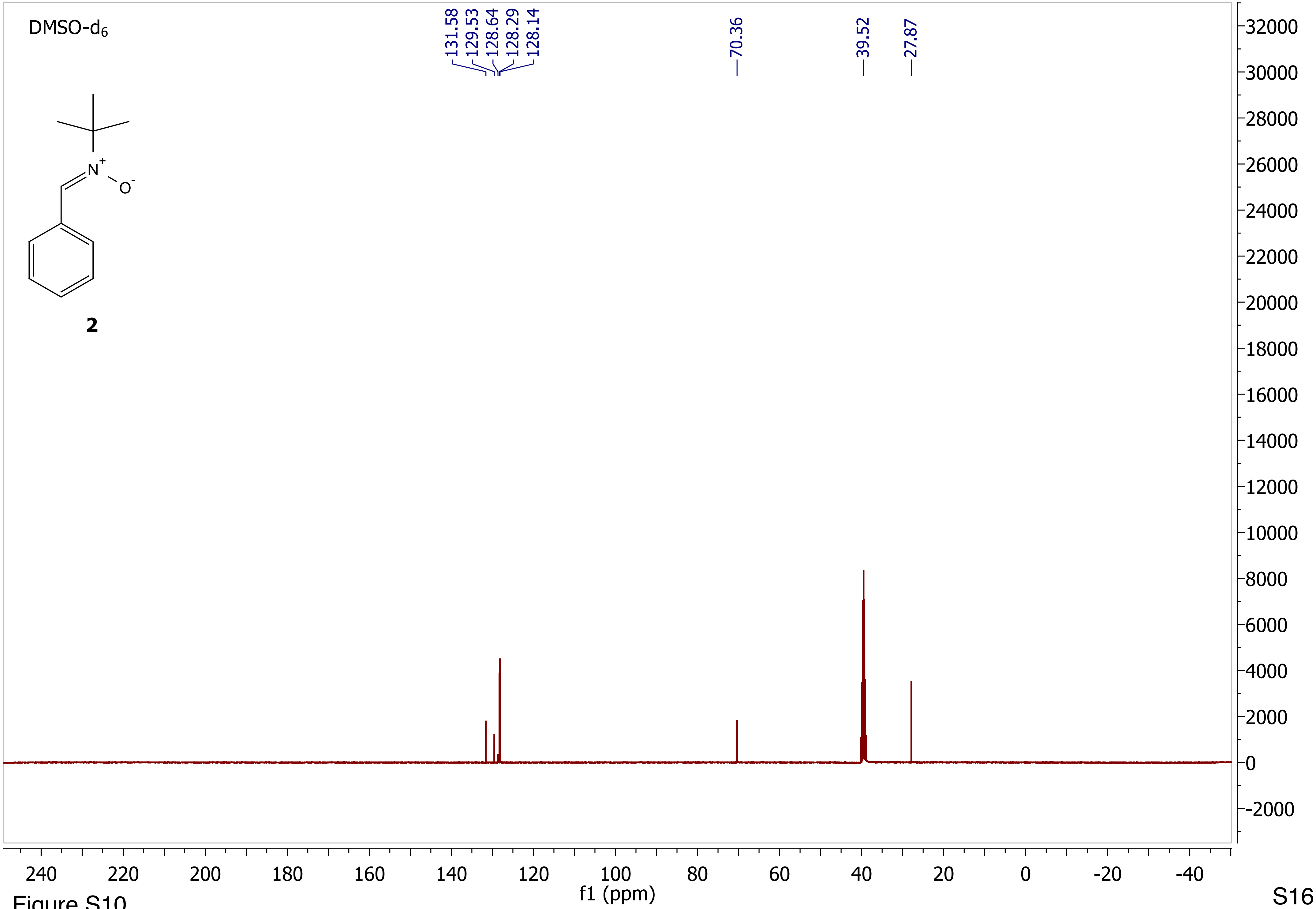

Figure S10
