# Supplementary material for: Safe and Sustainable by Design (SSbD) Synthesis of Food and Personal Care Ingredients by Mechanochemistry
Source: ChemSusChem. 2026 May 4;19(9):e202502491. doi: 10.1002/cssc.202502491 (PMC13139749; doi:10.1002/cssc.202502491)
Supplement: Supplementary file 1 — Supplementary Material [file CSSC-19-e202502491-s001.zip › 3_Ecoscale data_final_all compounds.pdf]

Synthesis in solution

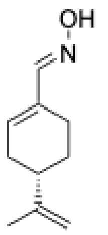

(S)-(-)-Perillartine (1)

THE ECOSCALE

Fast and transparent evaluation of organic preparations

Ecoscale calculator | Manual | Paper | Contact

Ecoscale calculator

Reagents

☐ Link

		identifier*	name	MF*	MW	density	purity*	ml	g	mmoles	equiv.
1	+ -		L(-)-Perillaldehyde	C10H14O	150.22056	0.97	100%	1.690722	1.64	10.917280563	1
2	+ -		Hydroxylamine hydrochloride	H3NO . HCl	69.49086		98%	0	1.04	14.666677027	1.3434368514
3	+ -		Sodium carbonate	CNa2O3	105.98874	2.53	99%	0.628458	1.59	14.851577629	1.3603733588
4	+ -		Water	H2O	18.01528	1	100%	20	20	1110.1687012	101.68912438
5	+ -		Ethyl acetate	C4H8O2	88.10632	0.902	100%	32.5	29.315	332.72301010	30.476729819
6	+ -		Cyclohexane	C6H12	84.16128	0.77	100%	17.5	13.475	160.10925689	14.665672092
7	+ -		Epsom salt	MgO4S	120.3626		100%	0	0.5	4.1541143179	0.3805081580

Products

identifier*:	name:	MF*:	MW:	g:	mmoles:	g theor:	yield:
	Perillartine	C10H15NO	165.2352	1.04	6.2940584088	1.803919	57.652300000

Conditions

Reagents

Name	mmoles	eq.	Bp	Hazard	Price
L(-)-Perillaldehyde	10.49	1	NaN		
Hydroxylamine hydrochloride	14.1	1.34			
Sodium carbonate	14.28	1.36	1600		
Water	1067.46	101.68			
Ethyl acetate	319.92	30.47	75		
Cyclohexane	153.95	14.66	81		
Epsom salt	3.99	0.38			

Yield	58		-21
Price / availability			-3
Safety			-30
Technical setup	Possible items: Common set-up; Instruments for controlled addition of chemicals; Unconventional activation technique	Selected items: Common set-up	0
Temperature / time	Possible items	Selected items	-1

	Possible items	Selected items	
	Room temperature, < 1h<br>Room temperature, < 24h<br>Heating, < 1h	Room temperature, < 24h	
Workup and purification	Sublimation<br>Liquid - liquid extraction or washing<br>Classical chromatography	Liquid - liquid extraction or washing<br>Crystallization and filtration<br>Removal of solvent with bp < 150°C	-4
EcoScale			41

^

Copyright 2006

The experimental protocol used for the calculations is indicated in:

T. Betke, P. Rommelmann, K. Oike, Y. Asano, H. Gröger, *Angew. Chem. Int. Ed.* 2017, **56**, 12361.
https://doi.org/10.1002/anie.201702952

Synthesis by Ball-milling : Table 1, entry 6

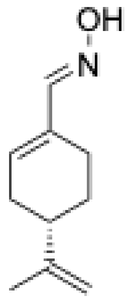

(*S*)-(-)-Perillartine (**1**)

Ecoscale calculator

Reagents

☑ Link

	identifier*	name	MF*	MW	density	purity*	ml	g	mmoles	equiv.
1		L(-)-Perillaldehyde	C10H14O	150.22056	0.97	100%	0.619466	0.600882	4	1
2		Hydroxylamine hydrochloride	H3NO . HCl	69.49086		100%	0	0.277963	4	1
3		Sodium bicarbonate	CHNaO3	84.00691		100%	0	0.336028	4	1
4		Water	H2O	18.01528	1	100%	12.5	12.5	693.85543827	173.46385956

Products

identifier*:	name:	MF*:	MW:	g:	mmoles:	g theor:	yield:
	Perillartine	C10H15NO	165.2352	0.6451	3.9041318072	0.660941	97.6033

Conditions

Reagents

Name	mmoles	eq.	Bp	Hazard	Price
L(-)-Perillaldehyde	6.2	1	NaN		
Hydroxylamine hydrochloride	6.2	1			
Sodium bicarbonate	6.2	1			
Water	1075.57	173.46			

Yield	98		-1
Price / availability			-3
Safety			-20
Technical setup	Possible items: Common set-up; Instruments for controlled addition of chemicals; Unconventional activation technique	Selected items: Common set-up	0
Temperature / time	Possible items: Room temperature, < 1h; Room temperature, < 24h; Heating, < 1h	Selected items: Room temperature, < 24h	-1
Workup and purification	Possible items: Crystallization and filtration; Removal of solvent with bp > 150°C; Solid phase extration	Selected items: Adding solvent; Simple filtration	0

EcoScale

75

Copyright 2006

Synthesis in solution

THE ECOSCALE
Fast and transparent evaluation of organic preparations

Ecoscale calculator | Manual | Paper | Contact

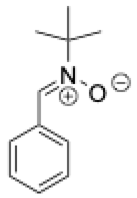

PBN (2)

Ecoscale calculator

Reagents

☐ Link

	identifier*	name	MF*	MW	density	purity*	ml	g	mmoles	equiv.
1		Benzaldehyde	C7H6O	106.12404	1.05	100%	0.020214	0.021225	0.2	1
2		N-(tert-Butyl)hydroxylamine hydrochloride	C4H12NOCl	125.59838		100%	0	0.02512	0.2	1
3		Pyrrolidine	C4H9N	71.12216	0.866	99%	0.01991	0.017242	0.24	1.2
4		Dichloromethane	CH2Cl2	84.93288	1.325	100%	0.61	0.80825	9.5163380777	47.581690388
5		Silicagel	O2Si	60.0843	2.13	100%	0.469484	1	16.643282854	83.216414271
6		Ethyl acetate	C4H8O2	88.10632	0.902	100%	5	4.51	51.188155401	255.94077700

Products

identifier*:	name:	MF*:	MW:	g:	mmoles:	g theor:	yield:
	N-tert-Butyl-alpha-phenylnitrone	C11H15NO	177.2462	0.0344	0.1940803244	0.035449	97.0408

Conditions

Reagents

Name	mmoles	eq.	Bp	Hazard	Price
Benzaldehyde	5.81	1	179		
N-(tert-Butyl)hydroxylamine hydrochloride	5.81	1			
Pyrrolidine	6.97	1.2	86		
Dichloromethane	276.63	47.58	39		
Silicagel	483.81	83.21			
Ethyl acetate	1488.02	255.94	75		

Yield	97			-1
Price / availability				-14
Safety				-20
Technical setup	Possible items: Common set-up; Instruments for controlled addition of chemicals; Unconventional activation technique		Selected items: Common set-up	0
Temperature / time	Possible items: Heating, > 1h; Cooling to 0°C; Cooling, < 0°C		Selected items: Room temperature, < 1h	0

Workup and purification

Possible items	Selected items	
Sublimation	Adding solvent	0
Liquid - liquid extraction or washing	Simple filtration	
Classical chromatography	Removal of solvent with bp < 150°C	

EcoScale 65

^

Copyright 2006

The experimental protocol used for the calculations is reported in:

S. Morales, F. G. Guijarro, I. Alonso, J. L. García Ruano and M. B. Cid, *ACS Catal.*, 2016, **6**, 84-91. https://doi.org/10.1021/acscatal.5b0172

THE ECOSCALE

Fast and transparent evaluation of organic preparations

Synthesis by ball-milling (Table 2, entry 6)

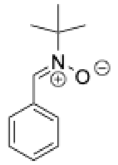

PBN (2)

Ecoscale calculator Manual Paper Contact

Ecoscale calculator

Reagents

☐ Link

		identifier*	name	MF*	MW	density	purity*	ml	g	mmoles	equiv.
1	+ -		Benzaldehyde	C7H6O	106.12404	1.05	100%	0.404282	0.424496	4	1
2	+ -		N-(tert-Butyl)hydroxylamine hydrochloride	C4H12NOCl	125.59838		100%	0	0.502394	4	1
3	+ -		Sodium bicarbonate	CHNaO3	84.00691		100%	0	0.336028	4	1
4	+ -		Water	H2O	18.01528	1	100%	12.5	12.5	693.85543827	173.46385956

Products

identifier*:	name:	MF*:	MW:	g:	mmoles:	g theor:	yield:
	N-tert-Butyl-alpha-phenylnitrone	C11H15NO	177.2462	0.461	2.6009020221	0.708985	65.022500000

Conditions

Reagents

Name	mmoles	eq.	Bp	Hazard	Price
Benzaldehyde	8.67	1	179		
N-(tert-Butyl)hydroxylamine hydrochloride	8.67	1			
Sodium bicarbonate	8.67	1			
Water	1505.1	173.46			

Yield: 65 — -17

Price / availability: -8

Safety: -5

Technical setup
Possible items: Common set-up; Instruments for controlled addition of chemicals; Unconventional activation technique
Selected items: Common set-up
0

Temperature / time
Possible items: Room temperature, < 1h; Room temperature, < 24h; Heating, < 1h
Selected items: Room temperature, < 24h
-1

Workup and purification
Possible items: Adding solvent; Simple filtration; Removal of solvent with bp < 150°C; Crystallization and filtration
Selected items: Adding solvent; Simple filtration
0

EcoScale	69	^

Copyright 2006

Synthesis by Ball-milling (literature)

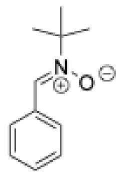

PBN (**2**)

THE ECOSCALE

Fast and transparent evaluation of organic preparations

Ecoscale calculator | Manual | Paper | Contact

Ecoscale calculator

Reagents

☑ Link

	identifier*	name	MF*	MW	density	purity*	ml	g	mmoles	equiv.
1		Benzaldehyde	C7H6O	106.12404	1.05	100%	0.050535	0.053062	0.5	1
2		N-(tert-Butyl)hydroxylamine hydrochloride	C4H12NOCl	125.59838		100%	0	0.062799	0.5	1
3		Sodium bicarbonate	CHNaO3	84.00691		100%	0	0.042003	0.5	1
4		Dichloromethane	CH2Cl2	84.93288	1.325	100%	4	5.3	62.402216903	124.80443380

Products

identifier*:	name:	MF*:	MW:	g:	mmoles:	g theor:	yield:
	N-tert-Butyl-alpha-phenylnitrone	C11H15NO	177.2462	0.088	0.4964845508	0.088623	99.297

Conditions

Reagents

Name	mmoles	eq.	Bp	Hazard	Price
Benzaldehyde	5.68	1	179		
N-(tert-Butyl)hydroxylamine hydrochloride	5.68	1			
Sodium bicarbonate	5.68	1			
Dichloromethane	709.11	124.8	39		

Yield: 99 — 0

Price / availability: -6

Safety: -5

Technical setup
Possible items: Common set-up; Instruments for controlled addition of chemicals; Unconventional activation technique
Selected items: Common set-up
0

Temperature / time
Possible items: Room temperature, < 1h; Room temperature, < 24h; Heating, < 1h
Selected items: Room temperature, < 24h
-1

Workup and purification
Possible items: Adding solvent; Simple filtration; Removal of solvent with bp < 150°C; Crystallization and filtration
Selected items: Adding solvent; Simple filtration
0

EcoScale 88 ^

Copyright 2006

The experimental protocol used for the calculations is reported in:

Colacino, E.; Nun, P.; Colacino, F.M.; Martinez, J.; Lamaty, F. *Tetrahedron* 2008, **64**, 5569-5576.
https://doi.org/10.1016/j.tet.2008.03.091
